# Acupuncture and stroke motor rehabilitation: a decade of evidence synthesis via systematic mapping (2015–2024)

**DOI:** 10.3389/fneur.2025.1647086

**Published:** 2025-09-25

**Authors:** Chao Ke, Zhuo Zhou, Mengzi Sun, Wenying Shi, Min Xu, Rusin Su, Yilin Zhou, Zhiwen Ouyang, Jiaxin Yin, Wei Zhang

**Affiliations:** The First Hospital of Hunan University of Chinese Medicine, Changsha, China

**Keywords:** stroke, motor dysfunction, acupuncture, randomised controlled trial, systematic mapping

## Abstract

**Background:**

Acupuncture has shown efficacy in the treatment of post-stroke motor impairment, for which it has newfound renown on the world stage. However, the knowledge base has not yet been reviewed in terms of international clinical trial design. This study systematically reviews the distribution and status of clinical research on acupuncture for treating post-stroke motor dysfunction within the last 10 years.

**Methods:**

We conducted a comprehensive search of four Chinese and three English databases from 1 January 2015 to 8 August 2024. We included randomised control trials (RCTs) with a control group. The study participants were diagnosed with post-stroke motor dysfunction according to clear diagnostic criteria regardless of nationality, race, sex, age, disease duration, or condition. The interventions included acupuncture administered alone or in combination with other acupuncture or non-acupuncture interventions. We included studies comparing different acupuncture therapies; acupuncture with sham treatments; acupuncture with other therapies; and compound effects. No restrictions were set on the type of outcome. Data were synthesised and visually analysed using Excel (2003), Office (2009), CiteSpace, SPSS Modeller 18.0, Hiplot, BioRender, and ChiPlot. Scientometric and visualisation analyses of the keywords were performed using CiteSpace. The Apriori algorithm in SPSS Modeller 18.0 was used to conduct an association analysis between acupoints and acupuncture therapies. Risk of bias assessments were performed using Cochrane RoB 2.0.

**Results:**

A total of 3,645 RCTs were included in the final review. Most studies were concentrated in China. Significant heterogeneity was observed in participants characteristics, intervention protocols, control groups, and clinical design. The RCTs showed a potentially high risk of bias owing to a lack of concealment methods and/or blinding.

**Conclusion:**

This study highlights evidence supporting the applicability of acupuncture as an effective and safe treatment for post-stroke motor dysfunction. The findings provide a useful reference for informing the design of future experimental and clinical studies, as well as guidance for clinical diagnosis and management.

## 1 Introduction

Stroke is one of the most common cerebrovascular disorders. It was the second leading cause of death and third leading cause of disability in 2019, according to the World Stroke Organization (WSO) (1, 2). The WSO estimates that there will be approximately 200 million stroke survivors globally by 2050 (3). Specifically, this chronic noncommunicable disease endangers public health in China (4). Stroke is also a major cause of long-term disability in humans; survivors often have varying degrees of motor, sensory, cognitive, speech, and swallowing impairments among other sequelae that limit rehabilitation and increase the risk of recurrence (5, 6). Motor dysfunction is the most common post-stroke symptom, with an incidence of up to 60%; it manifests as muscle weakness, abnormal posture and muscle tone, loss of coordination of trunk and limb movements, and impaired trunk control (7). These disabilities impact quality of life and impose substantial economic burdens on families and society.

Traditional Chinese medicine (TCM) has gained growing recognition around the world in recent years. Multiple studies and guidelines recognise the efficacy of acupuncture on motor dysfunction (8–11). In addition, functional magnetic resonance evidence suggests that acupuncture can promote the reorganization of neural plasticity in the brain by regulating brain network connections, which provides a new perspective for the modern scientific interpretation of acupuncture therapy (11, 12). However, no prior review has systematically synthesised the clinical trial evidence on acupuncture for post-stroke motor dysfunction. We conducted a comprehensive systematic review of randomised controlled trials (RCTs), domestically and internationally, to characterise the current state of research. Our review focused specifically on existing challenges and treatment strategies to provide a baseline reference for future clinical research, trial design, diagnosis, and management of acupuncture therapy following stroke.

## 2 Methods

### 2.1 Search strategy

This study is a systematic mapping review of randomised controlled trials (RCTs) on acupuncture for post-stroke motor dysfunction. Relevant medical journals in Chinese and English databases, such as the China National Knowledge Network, Wanfang Database, China BioMedical Database, Chonqing VIP Database, Cochrane Database of Systematic Reviews (CDSR), Cochrane Controlled Trials Register (CENTRAL), Embase, and PubMed, were searched from 1 January 2015 to 8 August 2024. Detailed search terms are provided in the Supplementary Materials 1.

### 2.2 Search terms

Type of study: We included full RCTs of acupuncture treatments for post-stroke motor dysfunction.

Type of participant: The study participants were diagnosed with post-stroke motor dysfunction according to clear diagnostic criteria regardless of nationality, race, sex, age, disease duration, or condition.

Type of intervention: Acupuncture therapy used alone or in combination with other acupuncture/non-acupuncture therapies.

Type of control: The control group is essential for validating the effects of acupuncture. We searched for studies comparing ① different acupuncture therapies; ② acupuncture with sham treatments; ③ acupuncture with other therapies; and ④ compound effects.

Type of outcome: No restrictions were set on the type of outcome.

### 2.3 Study selection and data extraction

Two authors independently screened the titles and abstracts after removing duplicates. Full-text articles were retrieved during further screening. Any disagreements were resolved by discussion; where no agreement was reached, the third author, Wei Zhang,served as an arbitrator to reduce potential bias. The extracted data included basic literature information (title, author, sources of literature, journal, journal grade, year of publication), patient (P) information (stroke categories, stroke phase, first episode, diagnostic criteria, paralysed pattern, paralysed body parts), intervention and control (I and C) information (total sample size, study design, intervention type, control type, acupuncture prescription), and outcome (O) information (outcome indicators and categories).

The acupoint prescription was pretreated as follows: (1) Acupoints were classified according to their names and locations in the 13th Five-Year National Teaching Material (Meridians and Acupoints). For unusual and unique acupoints (muscle/tendon points) not included in the teaching materials, the author’s record was used as the standard.(2) In these studies, two groups of acupoints were used alternately in the intervention group or, when there were two different acupuncture treatments in the intervention and control groups, they were divided into two groups of prescriptions. If repeated acupoints were used in different therapies in the same group, key terms were recorded for only one acupoint prescription. When multiple differentiated-compatible acupoints were reported, only the main points were selected. In cases involving a single syndrome type, the primary and supplementary acupoints were recorded. The acupoints were collated in a table for subsequent analyses.

### 2.4 Quality assessment

Risk of bias assessments were performed using the Cochrane Risk of Bias 2.0 (RoB 2.0) tool (13). The overall assessment was based on seven criteria classified as high, moderate, or low risk of bias. Two authors independently assessed the methodological quality of the 3,645 included studies. Any disagreements were resolved by discussion; where no agreement was reached, a third author served as an arbitrator.

### 2.5 Data analysis

The results are presented as text descriptions and charts. Data were synthesised and graphed using Excel 2003 (Microsoft Corporation, Redmond, WA, USA), Office 2009 (Microsoft Corporation), CiteSpace (version 6.3. R1), SPSS Modeller 18.0 (IBM SPSS, Armonk, NY, USA), Hiplot (https://hiplot.com.cn/), BioRender (https://app.biorender.com/), and ChiPlot (https://www.chiplot.online/). Scientometric and visualisation analyses of the keywords were performed using CiteSpace (version 6.3. R1). The Apriori algorithm in SPSS Modeller 18.0 was used to conduct an association analysis between acupoints and acupuncture therapies.

## 3 Results

**Figure 1:**
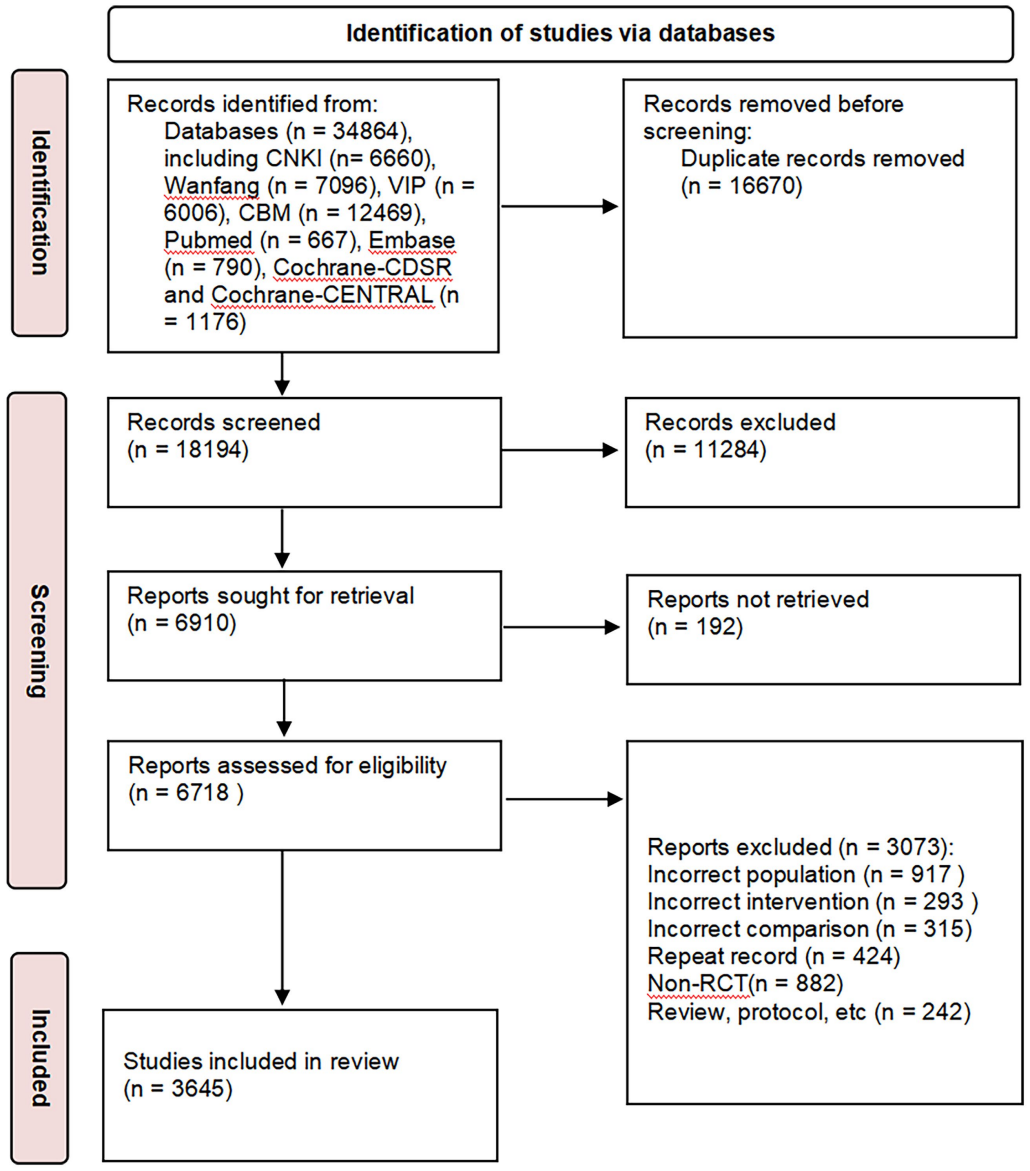
PRISMA workflow of literature selection. CNKI, China national knowledge infrastructure; CBM, China BioMedical database; CDSR, Cochrane database of systematic reviews; CENTRAL, Cochrane controlled trials register; RCT, randomised controlled trial.

In total, 34,863 literature items were retrieved (Figure 1). A total of 18,194 articles remained after duplicates were removed. From the title and abstract, 11,284 articles were removed for not meeting the inclusion/exclusion criteria, and 192 articles were not available. After reading the full text, 3,073 articles with repeated publications and PICO information that did not meet the requirements were excluded. A total of 3,645 articles were selected for the final analysis (14).

### 3.1 Basic characteristics of included studies

**Figure 2:**
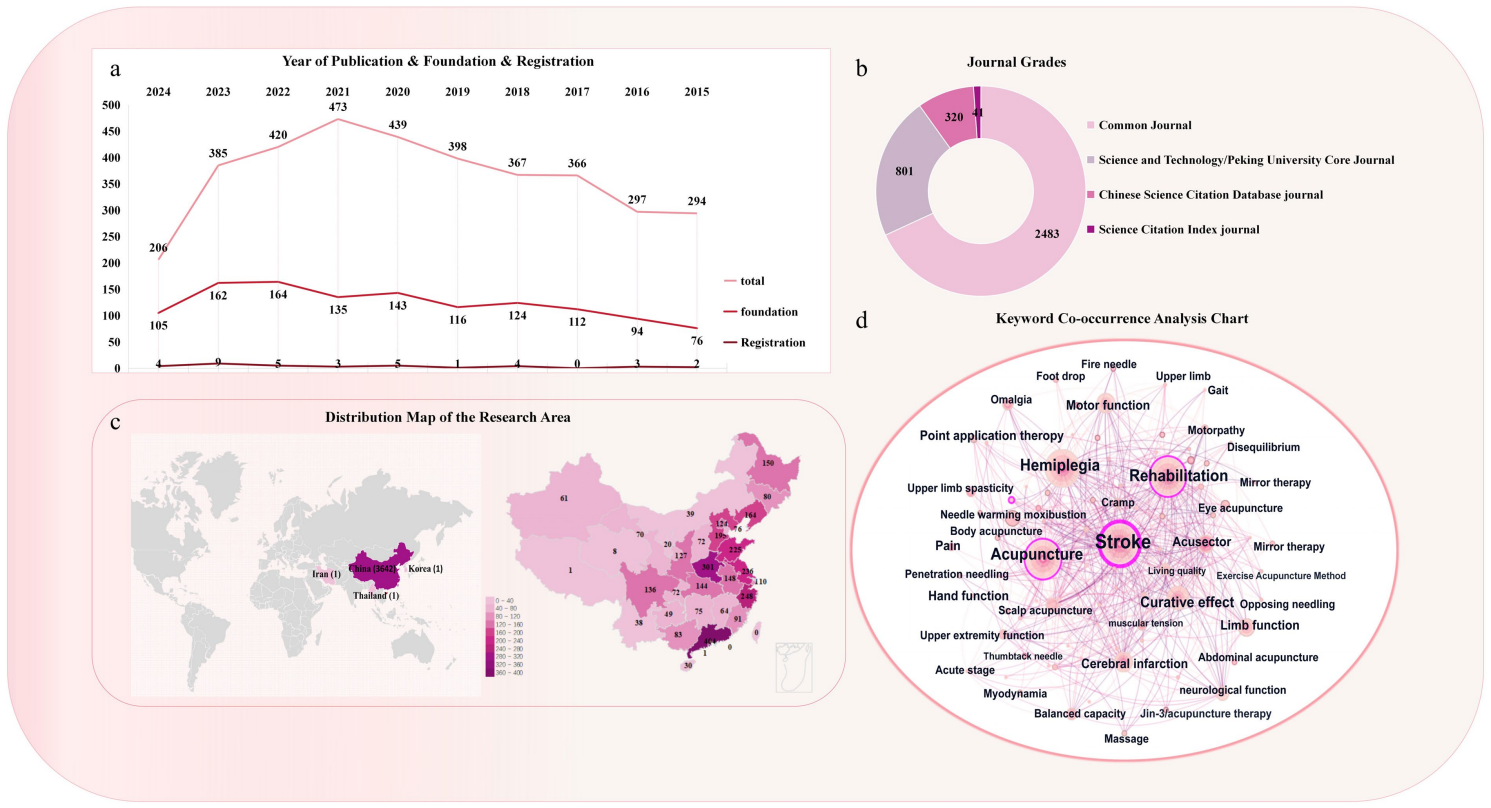
Basic characteristics of selected studies. **(a)** Annual publication, funding, and registration trends of the selected RCTs included in the research. **(b)** Distribution of journal grades included in the studies. **(c)** Distribution map of research areas of the included RCTs. **(d)** Keyword co-occurrence included in the research.

A total of 3,645 studies were published between 2015 and 2024. The number of published articles increased over time, peaking in 2021. The annual distribution, registration status, and funding support are shown (Figure 2a). A total of 3,645 articles were published in 398 different journals. The top three English journals were *Medicine*, *European Journal of Integrative Medicine*, and *Journal of Traditional Chinese Medicine*. The top three Chinese journals were *the Journal of Medicine & Health*, *Shanghai Journal of Acupuncture and Moxibustion*, and *Journal of Clinical Acupuncture and Moxibustion*. A total of 41/3,645 (1.12%) were Science Citation Index journals and 1,121/3,645 (30.75%) were Chinese Science Citation Database and Science and Technology/Peking University Core Journal sources (Figure 2b). The journal publishing area involved four countries; most studies were published in China (3,642/3,645, 99.92%). The highest number of studies were conducted in the Guangdong, Henan, and Zhejiang regions (Figure 2c).

The keyword co-occurrence analysis suggested that the most common disorders in the study population were stroke (665), hemiplegia (589), shoulder–hand syndrome (229), shoulder pain (66), foot varus (44), and foot drop (31). The most frequently used acupuncture interventions were acupuncture (631), electric acupuncture (188), head needle (114), warm needle (84), fire needle (44), moxibustion (30), and body acupuncture (28). Except for the acupuncture intervention, a high number of rehabilitation treatments (519) was observed. The efficacy indicators included transportation function (306), neurological function (153), living ability (141), quality of life (102), and balance (65) (Figure 2d).

### 3.2 Characteristics of included populations

**Figure 3:**
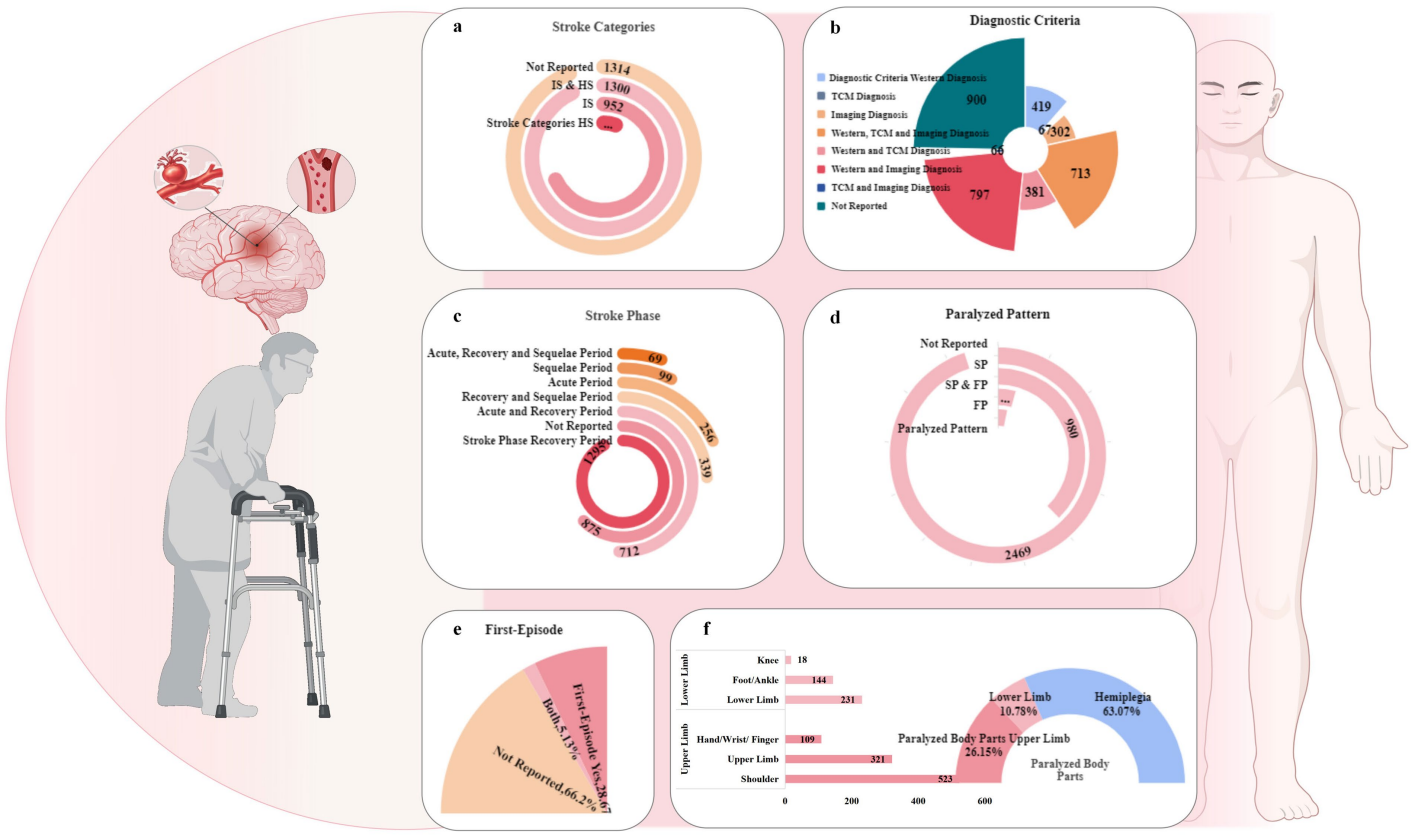
Population characteristics of the selected RCTs. **(a)** Stroke categories included in the research. **(b)** Reporting status of diagnostic criteria. **(c)** Stroke phases. **(d)** Paralysis patterns included in the studies. **(e)** First-episode status of the included RCTs. **(f)** Proportion of paralysed body parts. SP, spastic paralysis; FP, flaccid paralysis; IS, ischaemic stroke; HS, haemorrhagic stroke; TCM, traditional Chinese medicine.

Of the 3,645 studies, the sample size was a minimum of 10 and a maximum of 862. The sample sizes of the major trials ranged from 60 to 90 (1,775/3,645, 48.70%). The present review encompasses observations of upper limb hemiplegia, lower limb hemiplegia, and patients with hemiplegic as a whole. The patients recruited across the included studies varied in disease stage, stroke type, and movement disorders. Most patients were in periods of recovery (1,295/3,645, 35.53%), ischaemic stroke (IS; 952/3,645, 26.18%), and spastic paralysis (SP; 980/3,645, 26.89%). A total of 2,745 studies reported the sources of the diagnostic criteria: 797/3,645 (21.87%) reported Western medicine combined with imaging criteria, 713/3,645 (19.56%) reported Western medicine, TCM, and imaging criteria, and 419/3,645 (11.50%) Western medicine criteria (Figure 3). Detailed information is provided in Supplementary Materials 2.

### 3.3 Characteristics of intervention and control

**Figure 4:**
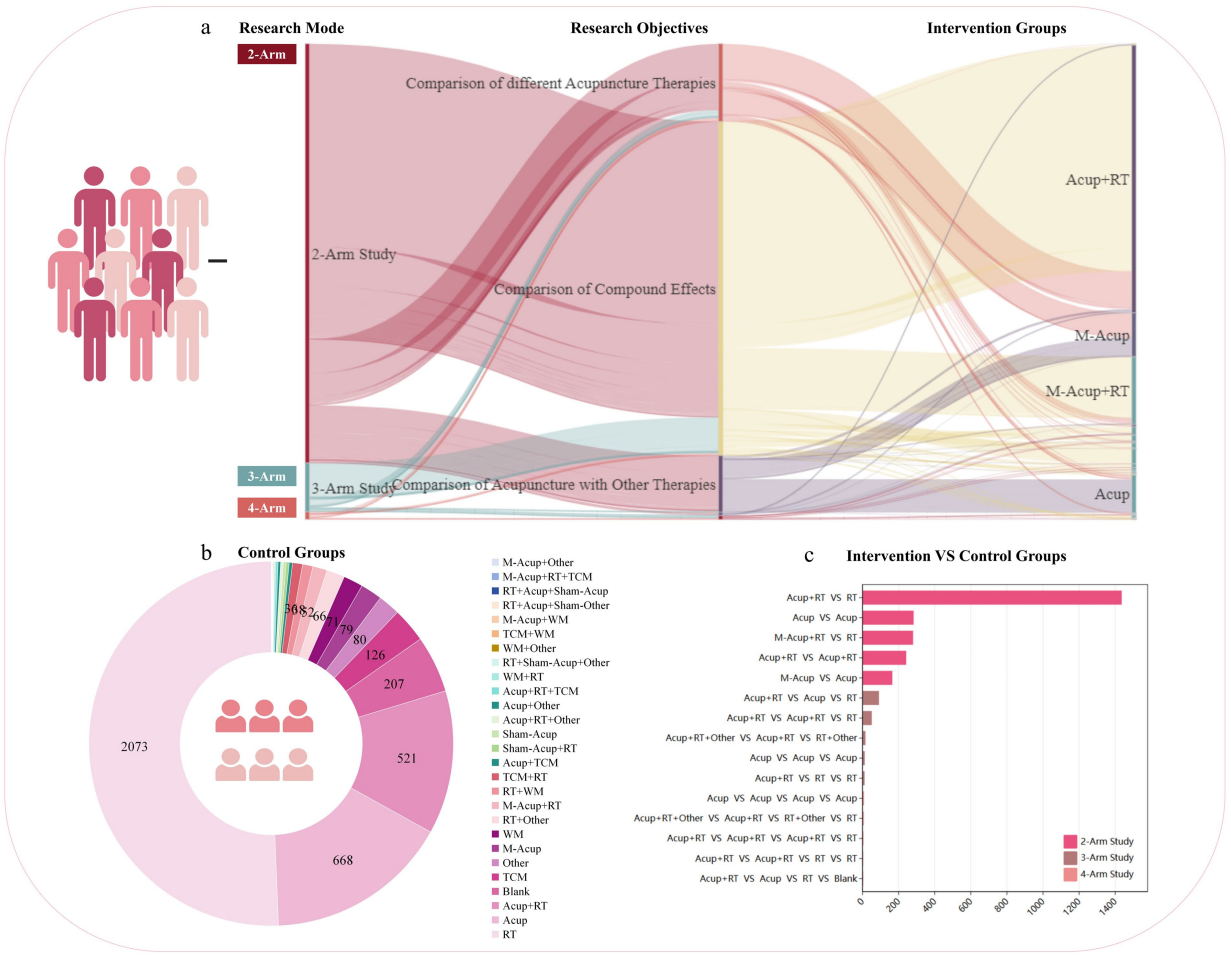
Intervention and control characteristics of selected RCTs. **(a)** Distribution of trial arms and intervention groups. **(b)** Distribution of control groups. **(c)** Top 5 trials (2-arm, 3-arm, and 4-arm designs). Acup, acupuncture; M-Acup, multi-acupuncture; RT, rehabilitation therapy; TCM, traditional Chinese medicine; WM, western medicine.

Of the 3,645 RCTs, 3,246 (89.05%) were 2-arm trials, 344 (9.44%) were 3-arm trials, 54 were 4-arm trials and one was a 5-arm trial. The studies included 2,360/3,645 (64.75%) comparisons of compound effects, 410/3,645 (5.76%) comparisons of acupuncture with other therapies, 23 comparisons of acupuncture with sham therapy, and 41 comparisons of different acupuncture therapies. In the intervention group, the top 3 treatments were acupuncture combined with rehabilitation, multi-acupuncture combined with rehabilitation, and acupuncture therapy (Figure 4a). In the control group, the top 3 treatments were rehabilitation, acupuncture, and acupuncture combined with rehabilitation therapy (Figure 4b). The designs of the top 5 interventions versus controls in the 2-arm, 3-arm, and 4-arm studies are shown in Figure 4c. The studies favoured acupuncture combined with rehabilitation versus rehabilitation alone.

**Figure 5:**
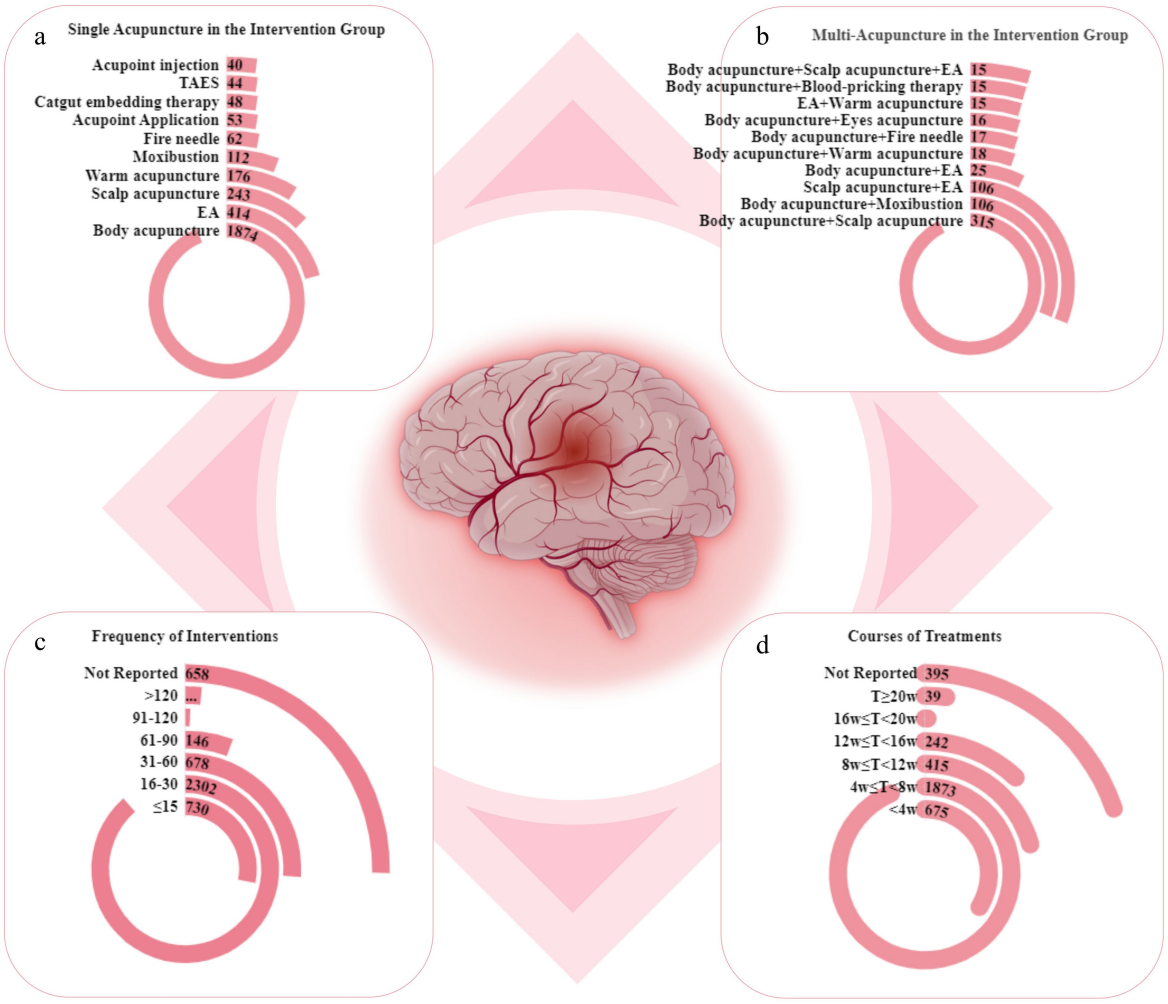
Intervention characteristics for acupuncture therapies in selected RCTs. **(a)** Top 10 acupuncture therapies. **(b)** Top 10 multi-acupuncture therapies. **(c)** Frequency of acupuncture sessions. **(d)** Treatment courses. TAES, transcutaneous acupoint electrical stimulation; EA, electroacupuncture.

We counted single and multiple acupuncture treatments separately and found that the top five acupuncture therapies were body acupuncture, electroacupuncture (EA), scalp acupuncture, warm acupuncture, and moxibustion (Figure 5a). The top five multi-acupuncture therapies were body acupuncture combined with scalp acupuncture, body acupuncture combined with moxibustion, scalp acupuncture combined with EA, body acupuncture combined with EA, and body acupuncture combined with warm acupuncture (Figure 5b). The total frequency of treatment in most studies was <30, which was related to the intervention method adopted by the institutes. When acupoint embedding or injection was used, the number of treatments was significantly lower than that for body injection interventions (Figure 5c). The treatment course for the acupuncture intervention was mostly between 4 and 8 weeks (1,873/3,645, 51.89%; Figure 5d). Detailed information is provided in Supplementary materials 2, 3.

### 3.4 Characteristics of acupoint prescriptions

Among the 3,645 RCTs, 4,510 acupoint prescriptions were identified, including 1,309 distinct acupoints, tenderness points, myofascial points, and muscle origin and insertion points. Regarding acupuncture interventions for post-stroke motor disorders, the 10 most frequently utilised acupoints were Hegu (LI4), Quchi (LI11), Zusanli (ST36), Sanyinjiao (SP6), Yanglingquan (GB34), Waiguan (SJ5), Shousanli (LI10), Jianyu (LI15), Neiguan (PC6), and Taichong (LR3).

**Figure 6:**
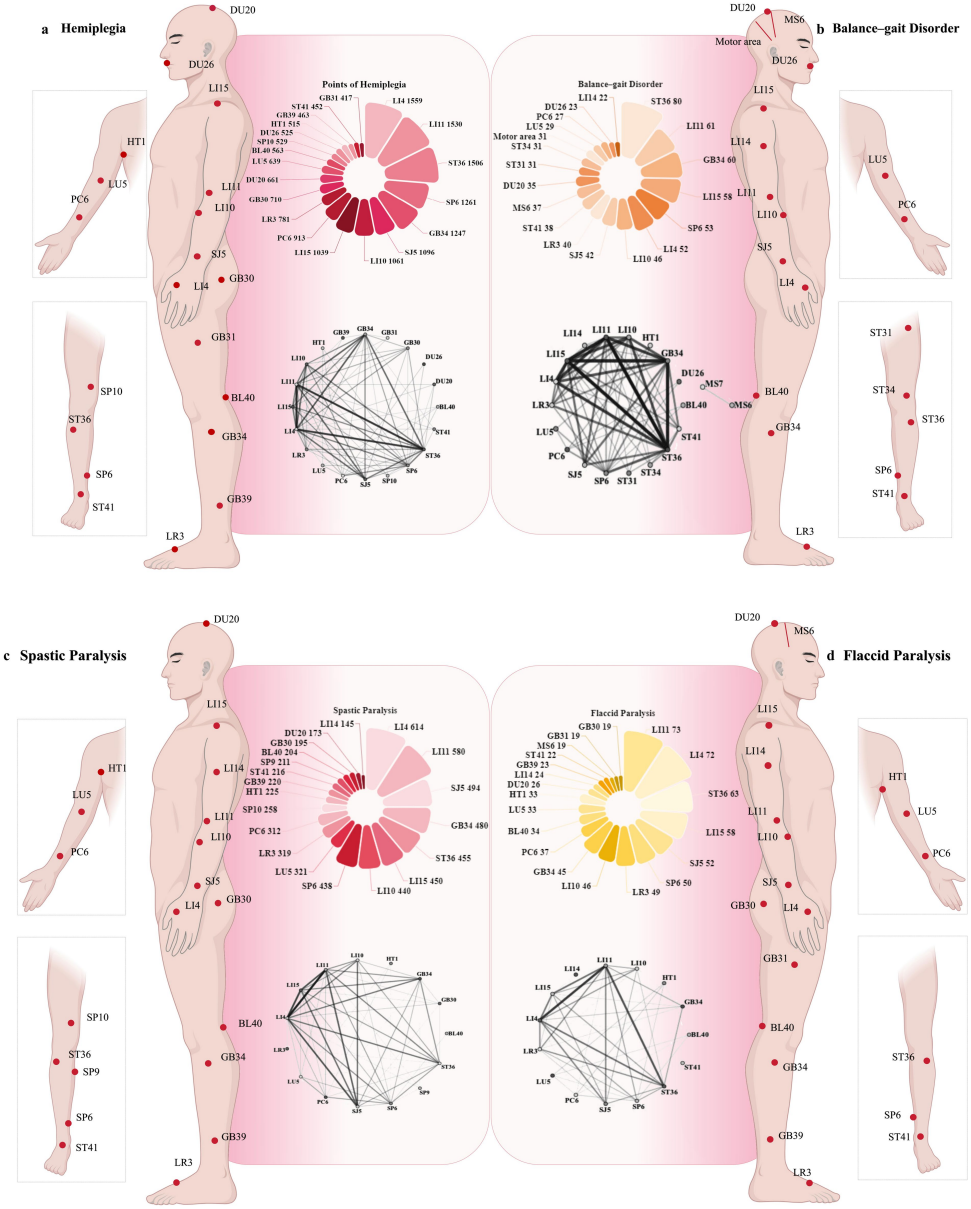
Acupoint characteristics of selected RCTs. **(a–d)** Acupoint prescription frequency links and top 20 acupoints from all acupoint prescriptions in people with hemiplegia, balance–gait disorders, flaccid paralysis, and spastic paralysis.

Apriori algorithm in SPSS Modeler 18.0 software was used to analyze the association of high-frequency acupoints, and the support degree was set ≥10% and the confidence degree was set ≥80% to make a network diagram. We analysed the acupoints of different paralysed body parts in post-stroke movement disorders according to onset and pathogenesis. Hegu (LI4), Quchi (LI11), Zusanli (ST36), Sanyinjiao (SP6), and Yanglingquan (GB34) were the five most frequently used acupoints. The highest frequency links between acupoints prescriptions were LI4–LI11 (1,249), LI11–ST36 (1,193), LI4–ST36 (1,169) (Figure 6a). Regarding balance–gait disorders, the most common acupoints were Zusanli (ST36), Quchi (LI11), Yanglingquan (GB34), Jianyu (LI15), and Sanyinjiao (SP6). The highest frequency links were LI11–ST36 (50), LI15–ST36 (49), and GB34–ST36 (48) (Figure 6b). In terms of paralysis mode, Hegu (LI4), Quchi (LI11), Waiguan (SJ5), Yanglingquan (GB34), and Zusanli (ST36) were the most popular acupuncture points for SP. The highest frequency links were LI4–LI11 (468), LI11–SJ5 (393), and LI4–SJ5 (375) (Figure 6c). Quchi (LI11), Hegu (LI4), Zusanli (ST36), Jianyu (LI15), and Waiguan (SJ5) were the most frequently used in flaccid paralysis (FP). The highest frequency links were LI4–LI11 (61), LI11–ST36 (55), and LI15–LI11 (49) (Figure 6d). Detailed information is provided in Supplementary Materials 5.

### 3.5 Characteristics of outcome indicators

**Figure 7:**
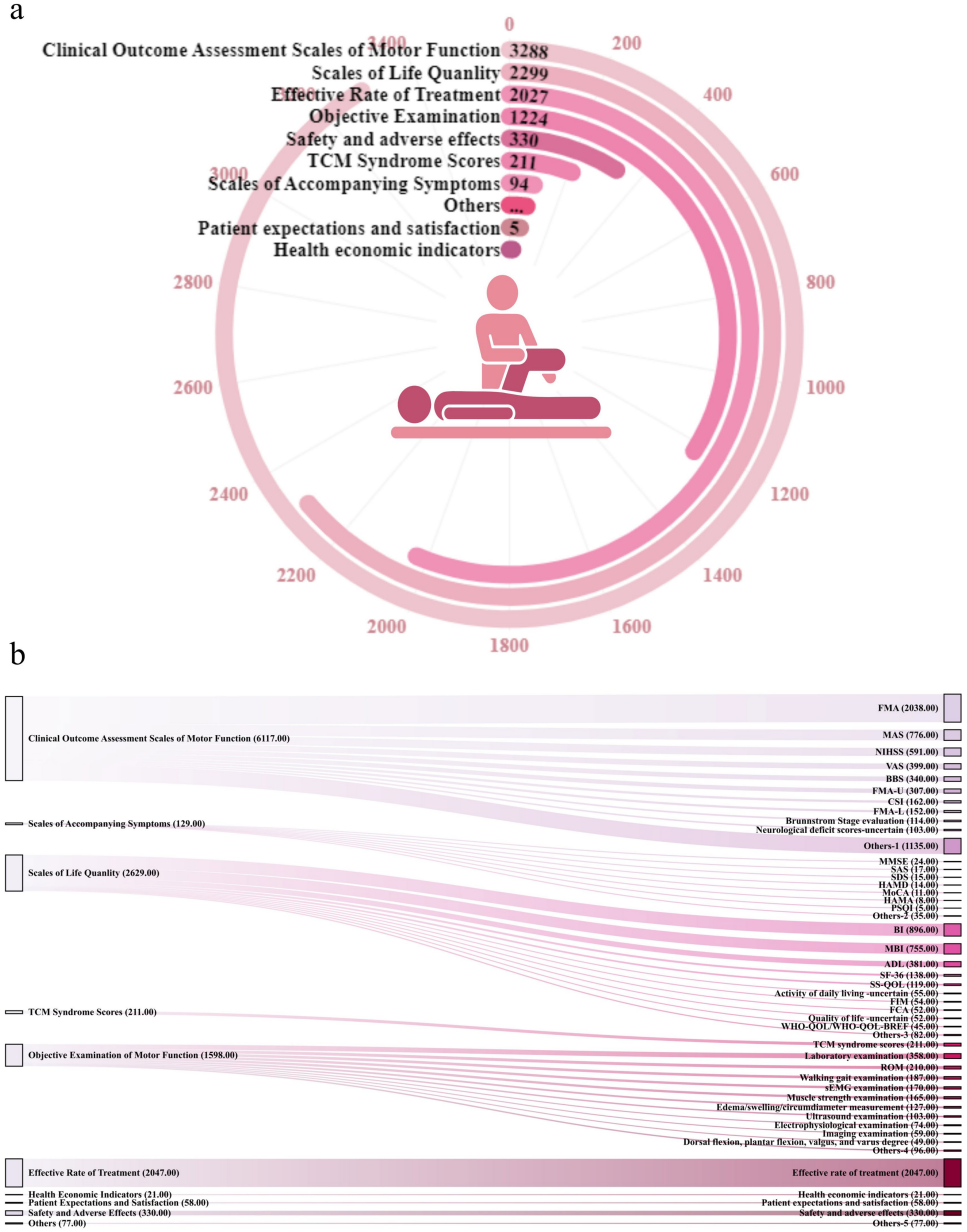
Outcome characteristics of selected RCTs. **(a)** Number of articles reported for each indicator category. **(b)** Morus chart on the left represents the eight categories, and that on the right represents the specific indicators under the categories. FMA, Fugl-Meyer motor assessment; MAS, Modified Ashworth Scale; NIHSS, National Institutes of Health Stroke Scale; VAS, Visual Analog Scale/Score; BBS, Berg Balance Scale; CSI, composite spasticity index; MMSE, mini-mental state examination; SAS, Self-rating Anxiety Scale score; SDS,self-rating depression scale; HAMD, Hamilton Depression Rating Scale; HAMA, Hamilton Anxiety Scale; MoCA, Montreal Cognitive Assessment Scale; PSQI, Pittsburgh Sleep Quality Index; BI, Barthel Index; MBI, Modified Barthel Index; ADL, activities of daily living; SF-36, Quality of Life-36; SS-QOL, stroke-specific quality of life; FIM, functional independence measure; FCA, functional capacity assessment; WHOQOL-BREF, World Health Organization Quality of Life Brief Scale; TCM, traditional Chinese medicine.

The outcomes were categorised into 10 groups (Figure 7): ① Clinical outcome assessment scales of motor function—assessing improvements in neurological deficits and motor function—were reported in 3,288/3,645 (90.21%) RCTs, with a total frequency of 6,117. ② Scales of accompanying symptoms—assessing enhancements in mental health, sleep, mood, cognition, swallowing, and other related symptoms using various scales—were reported in 94/3,645 (25.79%) RCTs, with a total frequency of 129. ③ Scales of life quality—assessing improvements in overall quality of life using various scales—were reported in 2,299/3,645 RCTs (63.07%), with a total frequency of 2,629. ④ TCM syndrome scores were reported in 211/3,645 (5.79%) RCTs, with a total frequency of 211. ⑤ Objective examination of motor function—including laboratory tests, imaging, and ultrasound examinations—was reported in 1,224/3,645 (33.58%) RCTs, with a total frequency of 1,598. ⑥ Effective rate of treatment—including effective, cure, and improvement rates—was reported in 2,027/3,645 (55.61%) RCTs, with a total frequency of 2,047. ⑦ Safety and adverse effects were reported in 330/3,645 (9.05%) RCTs, with a total frequency of 330. ⑧ Health economic indicators were reported in 21/3,645 (0.8%) RCTs, with a total frequency of 21. ⑨ Patient expectations and satisfaction were reported in 58/3,645 (1.59%) RCTs, with a total frequency of 58. Other indicators were noted in 77/3,645 (2.11%) RCTs, with a total frequency of 77. ⑩ Others. Detailed information is provided in Supplementary materials 2, 4.

### 3.6 Quality assessment

**Figure 8:**
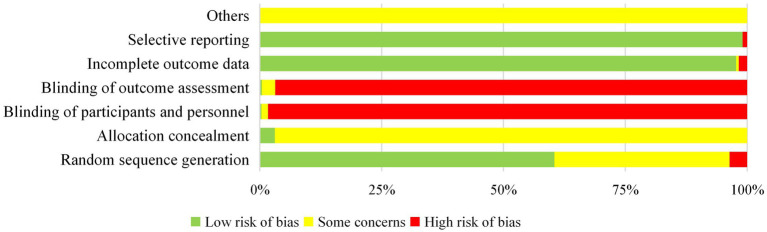
Cochrane bias risk analysis.

Few studies reported allocation concealment methods and blinding methods (Figure 8), indicating that the quality of the included studies was generally low.

## 4 Discussion

### 4.1 Literature status

Stroke is the most prevalent disease in China, causing various disabilities among more than two million new cases each year. Therefore, strengthening evidence-based practice and encouraging more translational studies are important to improve disease prognosis (15). Our study suggests that research on acupuncture for post-stroke motor dysfunction has continued to advance over the past 10 years, peaking in 2021 and declining slightly later. The novel coronavirus pandemic (2020 to 2022) significantly disrupted clinical research activities. Most research has been published in China—the birthplace of acupuncture medicine. There is a lack of high-quality research with international influence; however, the overall academic influence of journals requires careful consideration. From a geographical perspective, Guangdong, Henan, Zhejiang, Jiangsu, and Shandong saw outstanding rates of publication, among other regions, which may be related to the local climate, eating habits, scientific research level, and project funding. Notably, the RCT registration rate was <1%, indicating a critical need to improve awareness and implementation of clinical trial registration practices. The keyword analysis indicated that current research focused on people with hemiplegia, shoulder–hand syndrome, shoulder pain, foot varus, and foot drop, with stroke as the causative factor. EA, scalp acupuncture, warm acupuncture, fire acupuncture, moxibustion, and body acupuncture are research hotspots. Rehabilitation therapy was widely used to recover motor function. In terms of efficacy indicators, researchers have paid most attention to operational function, neurological function, quality of life, and balance ability, among other aspects. This was highly consistent with our PICO analysis, providing an important reference for the optimised design of future studies.

### 4.2 Included populations

Cerebral stroke is a common acute event that can be divided into two categories, IS and haemorrhagic stroke (HS). IS is the main subtype, accounting for 80–90% of stroke cases (16). Our study suggests that current motor dysfunction after stroke is mostly concentrated to IS cases, and patients tend to experience recovery 2 weeks to 6 months after onset. The recovery period is a key factor in the recovery of stroke patients, which directly affects their prognosis and quality of life. The American Stroke Association (ASA) recommends early rehabilitation for patients hospitalised with stroke to improve prognosis and reduce the risk of complications (17). Notably, in terms of abnormal movement patterns, spasticity is one of the most common complications in post-stroke motor dysfunction and usually occurs during the recovery period around 3 months after stroke (18). SP is a focus area and key challenge in current rehabilitation treatments. It is mainly characterised by speed-dependent muscle enhancement and increased tendon hyper-reflexes caused by the increased excitability of the stretch reflex. Moderate SP in stroke patients is associated with a lower risk of complications; however, severe spasticity may limit daily activities and seriously affect the recovery of motor function (19). Post-stroke spasticity can lead to a four-fold increase in medical costs, aggravating the economic burden of patients (20). Consequently, it is imperative to manage severe spasticity in patients with stroke. Prompt intervention is essential to mitigate disease progression, prevent degeneration, and inhibit further progression, consistent with TCM theory. In acupuncture intervention studies, researchers should not only rely on Western medicine and imaging diagnostics, but also TCM diagnostic criteria. Through the complementary application of traditional Chinese and Western medicine diagnostics, the benefits of TCM can be realised through comprehensive theoretical and practical guidance for the effective treatment of post-stroke motor dysfunction.

### 4.3 Intervention and control design

Impairment of motor function is one of the most serious consequences of stroke and remains a core focus and challenge in rehabilitation. As early as 1998, acupuncture was recommended by the World Health Organization as an alternative and complementary strategy for stroke treatment and for improving stroke care; acupuncture therapy was also favoured by clinical workers (21). Current acupuncture research is dominated by 2-arm RCTs, and less so by 3- and 4-arm RCTs. This may be because 2-arm RCTs are simpler, more efficient, more cost-effective, and more suitable for single intervention comparison, whereas 3- and 4-arm RCTs are more informative but also more complex, specialised, and expensive. In terms of study design, most studies have concentrated on comparisons of acupuncture combined with other therapies versus other therapies, and group design has focused on acupuncture versus other therapies. There were insufficient studies comparing acupuncture with placebo/sham acupuncture, which may be due to challenges related to study design and sham acupuncture implementation. In addition, there have been few comparisons of different acupuncture therapies, with few studies focusing on the optimisation of internal acupuncture schemes, such as special acupuncture methods, acupuncture frequency, acupuncture time, stimulation amount, acupoint selection, and compatibility, requiring further investigation. Some studies have investigated the timing of acupuncture in the treatment of post-stroke motor dysfunction and the influence of disease severity and stage, though further research is needed. Based on these findings, trials can be optimised in terms of the acupuncture treatment selection and intervention timing to provide evidence-based clinical treatments with improved efficacy within a given disease context and promote the standardised application of acupuncture and moxibustion globally.

Previous acupuncture studies often used acupuncture in combination with rehabilitation. An increasing number of clinical studies have shown that acupuncture and rehabilitation demonstrate a mutually reinforcing/synergistic relationship in improving prognosis in post-stroke motor dysfunction (22), by coordinating the central nervous system plasticity, neurotransmitters and neurotrophic factors, inhibiting abnormal reflexes and other ways (23–25). In terms of acupuncture therapy, body acupuncture, head acupuncture, EA, moxibustion, warm acupuncture, fire acupuncture and other treatment methods have been reported. The acupuncture system has been widely used in motor disorders, such as balance needle, wrist and ankle needle, abdominal needle, and eye needle. This reflects the diversification and integration of treatments for post-stroke motor dysfunction. Importantly, although the current study encompassed a wide range of comprehensive treatment modalities, it was challenging to ascertain the relative effectiveness of the specific interventions. This also underscores the deficiency in economic health indicators. The design of control groups is typically aligned with the research objectives and often includes rehabilitation, acupuncture alone, or a combination of acupuncture and rehabilitation. Future studies should clarify the efficacy weights of different treatments and employ health economic indicators to optimise treatment options and fully leverage the advantages of acupuncture interventions in the treatment of post-stroke movement disorders.

### 4.4 Acupuncture point prescription

Hegu (LI4), Quchi (LI11), Zusanli (ST36), Sanyinjiao (SP6), Yanglingquan (GB34), Waiguan (SJ5), Shousanli (LI10), Jianyu (LI15), Neiguan (PC6), and Taichong (LR3) are commonly used acupoints for post-stroke movement disorders. The selection of these acupoints reflects the principles of TCM, which includes ‘selecting acupoints along the meridians’ and ‘combining local and distal points. In addition to traditional acupoints, trigger points and myofascial nodal points based on Western anatomical knowledge have also been effectively employed. This demonstrates the robust clinical potential of acupuncture as it continues to evolve through foundational knowledge and innovation. Although there is an overlap in the selection of acupoints for spastic and flaccid paralyses, researchers often adapt needling techniques (such as reinforcement and reduction, depth of insertion, and intensity of stimulation) to suit different disease states. This embodies the TCM philosophy of ‘treating the same disease with different methods.

Han et al. (26). found that the functional connectivity between the premotor cortex/assisted motor region and the ipsilateral longitudinal starting and endpoints was significantly enhanced after acupuncture at Yanglingquan (GB34), which could increase the communication between the cortices connected by impaired white matter bundles and improve motor dysfunction. This also reveals the mechanism of action of acupuncture in promoting functional reconstruction by regulating neural network plasticity. In TCM, wind, fire, phlegm, blood stasis, and deficiency are considered the main pathogenic factors of stroke. The disease mainly affects the brain, heart, liver, spleen, kidney, and other viscera; thus, the treatment should aim to promote blood circulation, unblock meridians, and revitalise brain function. The Du, hand-and-foot Yangming, and foot Shaoyang gallbladder meridians are commonly used. Frequently selected acupoints include those on the Governor Vessel [such as Baihui (DU20) and Fengfu (DU16)], Yangming channels of the hand and foot [e.g. Hegu (LI4), Quchi (LI11), and Zusanli (ST36)], and the Gallbladder Channel of Foot-Shaoyang (e.g., Yanglingquan GB34). This approach emphasises the applicability of local acupoint selection to unblock the meridians and activate collaterals and distal acupoint selection to regulate the internal organs, fully demonstrating the root-and-branch characteristics of TCM.

### 4.5 Outcome indicators

The selection of appropriate outcomes is critical to the success of a study and important for improving reporting standards and research quality. The rationality of the selected outcomes should be consistent with the factors under investigation. An unsuitable choice may compromise the reliability and efficacy of acupuncture. Therefore, it is useful to standardise the outcome measures of acupuncture for post-stroke motor dysfunction in clinical studies. Among the most widely used outcome indicators were the clinical outcome assessment scales of motor function, scales of life quality, effective rate of treatment, and objective examination. There are few reports on indicators such as scales of accompanying symptoms, safety and adverse effects, TCM syndrome type, health economic indicators, safety evaluation, patient expectations, and satisfaction.

The clinical outcome assessment scales of motor function include multidimensional neurological deficit, motor function, spasticity/muscular tension systems, balance, and functional mobility scores. Although response efficiency is widely used, its definition criteria are not standardised, and some studies do not provide specific evaluation indicators. This ambiguity may introduce bias into trial conclusions. In recent years, a working group focused on core outcome measures in effectiveness trials recommended that the core indicator set for TCM should emphasise its specific characteristics, such as TCM syndrome indicators (27). However, most of these indicators are subjective, and their connotations are unclear; therefore, a more systematic and standardised evaluation system is urgently needed. Moreover, although health economic indicators can inform policy decision-making, standardised evaluation criteria are lacking, and reporting rates remain low. Acupuncture therapy has unique advantages in terms of few adverse reactions, high patient expectations, and high satisfaction; however, the reporting rate is limited, and the sample size in existing studies are generally small, requiring further large-scale verification.

The International Classification of Functioning, Disability, and Health (ICF) is a widely used framework for describing patient function, disability, and health from a bio-psychosocial perspective (28). Restoration of motor function is crucial for the social reintegration of patients with stroke. We aimed to analyse treatment progression from the physical to living levels, and ultimately the participation level, in accordance with the ICF framework; however, these major indicators in stroke still lack clear and standardised definitions. Particularly, there has been limited focus on the development and application of participation-level indicators. Clinical research has paid limited attention to the social participation abilities of patients with post-stroke motor dysfunction. The levels of societal reintegration, improvement in quality of life, and enhanced social adaptability require further investigation. Future research should employ participation-level indicators to comprehensively evaluate the long-term effects of acupuncture therapy on functional recovery and social reintegration and promote the transition of stroke rehabilitation from functional improvements to holistic social participation.

### 4.6 Methodological quality

The methodological quality of existing RCTs was relatively low, primarily owing to insufficient descriptions of allocation concealment methods, lack of blinding, and challenges associated with implementing double-blind designs. Key areas for improvement include optimising randomisation and allocation concealment schemes, exploring blinding designs suitable for the unique characteristics of acupuncture, and enhancing standardised reporting. At the same time, priority should be given to prospective registration, the use of core outcome index sets, and more large-scale multinational cooperative trials to improve the universality and methodological rigor of evidence. These efforts will strengthen the reliability and scientific rigor of applying acupuncture in the treatment of stroke and its complications.

### 4.7 Strengths and limitations

This systematic review presents the status of clinical research on acupuncture for the treatment of post-stroke motor dysfunction over the past decade for the first time. This study offers a useful reference for future research and trial design, provides guidance for clinical diagnosis and evaluation, and provides strong evidence on the applicability of acupuncture therapy. The review comprises a relatively large number of studies with a comprehensive scope. Our focus was on characterising RCTs from the past decade rather than conducting a comprehensive review from the inception of each relevant database. Despite the implementation of rigorous retrieval strategies and exclusion criteria, some relevant studies may have been inadvertently omitted. Furthermore, the scope of the database was confined to literature published in Chinese and English, which may introduce some bias.

## 5 Conclusion

This study provides strong evidence supporting the applicability of acupuncture as a safe and effective treatment for post-stroke motor dysfunction, but future RCTs must improve blinding, registration, and global representation. The findings provide a useful reference for informing the design of future experimental and clinical studies, as well as guidance for clinical diagnosis and management.

## Data Availability

The original contributions presented in the study are included in the article/Supplementary material, further inquiries can be directed to the corresponding author/s.

## Glossary

RCTs: Randomised Control Trials
WSO: World Stroke Organization
CDSR: Cochrane Database of Systematic Reviews
CENTRAL: Cochrane Controlled Trials Register
P: Patient
O: Outcome; I and C, intervention and Control
SP: Spastic Paralysis
FP: Flaccid Paralysis
IS: Ischaemic Stroke
HS: Haemorrhagic Stroke
TCM: Traditional Chinese medicine
Acup: Acupuncture
M-Acup: Multi-Acupuncture
RT: Rehabilitation Therapy
WM: Western Medicine
TAES: Transcutaneous Acupoint Electrical Stimulation
EA: Electroacupuncture
FMA: Fugl-Meyer Motor Assessment
MAS: Modified Ashworth Scale
NIHSS: National Institutes of Health Stroke Scale
VAS: Visual Analog Scale/Score
BBS: Berg Balance Scale
CSI: Composite Spasticity Index
MMSE: Mini-Mental State Examination
SAS: Self-Rating Anxiety Scale Score
SDS: Self-Rating Depression Scale
HAMD: Hamilton Depression Rating Scale
HAMA: Hamilton Anxiety Scale
MoCA: Montreal Cognitive Assessment Scale
PSQI: Pittsburgh Sleep Quality Index
BI: Barthel Index
MBI: Modified Barthel Index
ADL: Activities of Daily Living
SF-36: Quality of Life-36
SS-QOL: Stroke-Specific Quality of Life
FIM: Functional Independence Measure
FCA: Functional Capacity Assessment
WHO-QOL-BREF: World Health Organization Quality of Life Brief Scale
