# Supplementary material for: Acupuncture and stroke motor rehabilitation: a decade of evidence synthesis via systematic mapping (2015–2024)
Source: Front Neurol. 2025 Sep 25;16:1647086. doi: 10.3389/fneur.2025.1647086 (PMC12511885; doi:10.3389/fneur.2025.1647086)
Supplement: Supplementary file 1 [file Table_1.DOCX]

**Supplementary File 1. Search strategies of each database.**

Time：2015.01.01-2024.08.08

We searched seven language databases databases: MEDLINE (Pubmed), Embase, Cochrane Library, CNKI, Wanfang Data, Chongqing VIP, Sinomed to retrieve relevant studies.

**Pubmed——667**


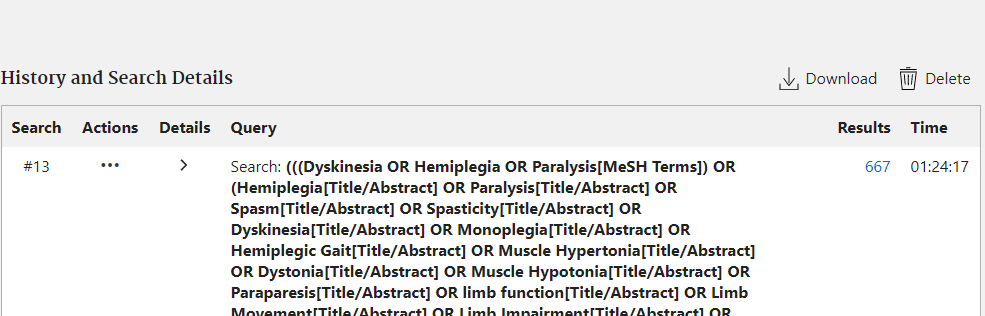


#1 Acupuncture OR Acupuncture Therapy OR Moxibustion[MeSH Terms]

#2 Acupuncture[Title/Abstract] OR Acupuncture Therapy[Title/Abstract] OR Acupuncture Treatment[Title/Abstract] OR Treatment, Acupuncture[Title/Abstract] OR Therapy, Acupuncture[Title/Abstract] OR Manual Acupuncture[Title/Abstract] OR Acupuncture-moxibustion[Title/Abstract] OR Moxibustion[Title/Abstract] OR Moxabustion[Title/Abstract] OR Warm needle[Title/Abstract] OR Warm Acupuncture[Title/Abstract] OR Thermoacupuncture[Title/Abstract] OR Electroacupuncture[Title/Abstract] OR Electro-acupuncture[Title/Abstract] OR electric acupuncture[Title/Abstract] OR Acupuncture Point[Title/Abstract] OR Point, Acupuncture[Title/Abstract] OR Acupoint[Title/Abstract] OR Pharmacopuncture[Title/Abstract] OR Pharmacoacupuncture Treatment[Title/Abstract] OR Treatment, Pharmacoacupuncture[Title/Abstract] OR Pharmacoacupuncture Therapy[Title/Abstract] OR Therapy, Pharmacoacupuncture[Title/Abstract] OR Acupoint injection[Title/Abstract] OR acupuncture injection[Title/Abstract] OR pharmaco-acupuncture[Title/Abstract] OR auricular needle[Title/Abstract] OR Acupunctures, Ear[Title/Abstract] OR Ear Acupuncture[Title/Abstract] OR Ear needle[Title/Abstract] OR earlobe acupuncture[Title/Abstract] OR Auricular Acupuncture[Title/Abstract] OR Auriculotherapy[Title/Abstract] OR Acupuncture, Auricular[Title/Abstract] OR auricular plaster therapy[Title/Abstract] OR auricular point sticking[Title/Abstract] OR Auricular pressure[Title/Abstract] OR Fire needle[Title/Abstract] OR Fire acupuncture[Title/Abstract] OR Acupoint catgut embedding[Title/Abstract] OR Scalp acupuncture[Title/Abstract] OR Scalp needle[Title/Abstract] OR Scalp electroacupuncture[Title/Abstract] OR scalp stimulation[Title/Abstract] OR Eye needle[Title/Abstract] OR Eye acupuncture[Title/Abstract] OR Abdominal acupuncture[Title/Abstract] OR Abdominal needle[Title/Abstract] OR filiform needle[Title/Abstract] OR silver needle[Title/Abstract] OR three-edged needle[Title/Abstract] OR intradermal needle[Title/Abstract] OR Point application[Title/Abstract] OR needle-embedding[Title/Abstract] OR Catgut Embedding[Title/Abstract] OR pricking therapy[Title/Abstract] OR point injection[Title/Abstract] OR Skin Acupuncture[Title/Abstract] OR transcutanclus electrical acupoint stimulation[Title/Abstract] OR TEAS[Title/Abstract] OR electrical acupoint stimulation[Title/Abstract] OR Acupuncture Point[Title/Abstract] OR Acupoint[Title/Abstract]

#3 #1 OR #2

#4 Stroke OR Apoplexy[MeSH Terms]

#5 "stroke*"[Title/Abstract] OR "Apoplexy"[Title/Abstract] OR "cerebrovascular apoplexy"[Title/Abstract] OR "apoplexy cerebrovascular"[Title/Abstract] OR "cerebrovascular stroke*"[Title/Abstract] OR "cerebral stroke*"[Title/Abstract] OR "acute stroke*"[Title/Abstract] OR "embolic stroke*"[Title/Abstract] OR "wake up stroke*"[Title/Abstract] OR "ischemic stroke*"[Title/Abstract] OR "thrombotic stroke*"[Title/Abstract] OR "brain ischemia"[Title/Abstract] OR "stroke* cerebrovascular"[Title/Abstract] OR "stroke* cerebral"[Title/Abstract] OR "stroke* acute"[Title/Abstract] OR "brain infarction"[Title/Abstract] OR "cerebral infarct*"[Title/Abstract] OR "intracerebral hemorrhage*"[Title/Abstract] OR "cerebral hemorrhage*"[Title/Abstract] OR "cerebral brain hemorrhage*"[Title/Abstract] OR "hemorrhagic stroke*"[Title/Abstract] OR "hypertensive intracerebral hemorrhage*"[Title/Abstract] OR ("hemorrhage*"[All Fields] AND "Cerebrum"[Title/Abstract]) OR (("Cerebrum"[MeSH Terms] OR "Cerebrum"[All Fields] OR "cerebrums"[All Fields]) AND "hemorrhage*"[Title/Abstract]) OR "cerebral parenchymal hemorrhage*"[Title/Abstract] OR ("hemorrhage*"[All Fields] AND "cerebral parenchymal"[Title/Abstract]) OR "parenchymal hemorrhage* cerebral"[Title/Abstract] OR "hemorrhage* intracerebral"[Title/Abstract] OR "hemorrhage* cerebral"[Title/Abstract] OR "cerebral hemorrhage*"[Title/Abstract] OR "brain hemorrhage* cerebral"[Title/Abstract] OR ("hemorrhage*"[All Fields] AND "cerebral brain"[Title/Abstract]) OR "cerebrovascular accident*"[Title/Abstract] OR "acute cerebrovascular accident*"[Title/Abstract] OR "cva"[Title/Abstract] OR "vascular accident* brain"[Title/Abstract] OR "brain vascular accident*"[Title/Abstract]

#6 #4 OR #5

#7 Dyskinesia OR Hemiplegia OR Paralysis[MeSH Terms]

#8 Hemiplegia[Title/Abstract] OR Paralysis[Title/Abstract] OR Spasm[Title/Abstract] OR Spasticity[Title/Abstract] OR Dyskinesia[Title/Abstract] OR Monoplegia[Title/Abstract] OR Hemiplegic Gait[Title/Abstract] OR Muscle Hypertonia[Title/Abstract] OR Dystonia[Title/Abstract] OR Muscle Hypotonia[Title/Abstract] OR Paraparesis[Title/Abstract] OR limb function[Title/Abstract] OR Limb Movement[Title/Abstract] OR Limb Impairment[Title/Abstract] OR motor function[Title/Abstract] OR Motor Dysfunction[Title/Abstract] OR Motor Impairment[Title/Abstract] OR Gait Disorder[Title/Abstract] OR Gait Dysfunction[Title/Abstract] OR Limb[Title/Abstract] OR Gait[Title/Abstract] OR motor[Title/Abstract] OR Lower limb[Title/Abstract] OR lower extremities[Title/Abstract] OR upper extremity[Title/Abstract] OR upper limb[Title/Abstract] OR leg[Title/Abstract] OR digit[Title/Abstract] OR toe[Title/Abstract] OR knee[Title/Abstract] OR ankle[Title/Abstract] OR foot[Title/Abstract] OR thigh[Title/Abstract] OR Lower extremity[Title/Abstract] OR shoulder[Title/Abstract] OR arm[Title/Abstract] OR forearm[Title/Abstract] OR wrist[Title/Abstract] OR hand[Title/Abstract] OR Hip joint[Title/Abstract] OR Balance[Title/Abstract]

#9 #7 OR #8

#10 #3 AND #6 AND #9

**Cochrane-1176**

**
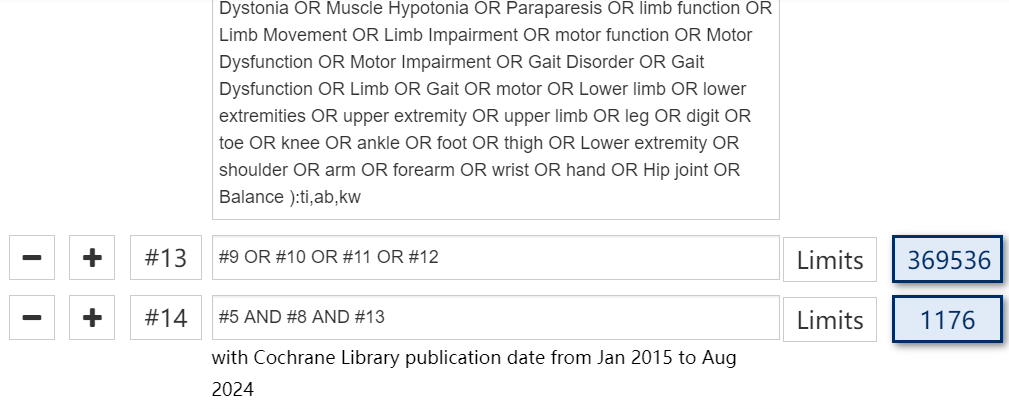
**

#1 MeSH descriptor: [Acupuncture] explode all trees

#2 MeSH descriptor: [Acupuncture Therapy] explode all trees

#3 MeSH descriptor: [Moxibustion] explode all trees

#4(Acupuncture OR Acupuncture Therapy OR Acupuncture Treatment OR Treatment, Acupuncture OR Therapy, Acupuncture OR Manual Acupuncture OR Acupuncture-moxibustion OR Moxibustion OR Moxabustion OR Warm needle OR Warm Acupuncture OR Thermoacupuncture OR Electroacupuncture OR Electro-acupuncture OR electric acupuncture OR Acupuncture Point OR Point, Acupuncture OR Acupoint OR Pharmacopuncture OR Pharmacoacupuncture Treatment OR Treatment, Pharmacoacupuncture OR Pharmacoacupuncture Therapy OR Therapy, Pharmacoacupuncture OR Acupoint injection OR acupuncture injection OR pharmaco-acupuncture OR auricular needle OR Acupunctures, Ear OR Ear Acupuncture OR Ear needle OR earlobe acupuncture OR Auricular Acupuncture OR Auriculotherapy OR Acupuncture, Auricular OR auricular plaster therapy OR auricular point sticking OR Auricular pressure OR Fire needle OR Fire acupuncture OR Acupoint catgut embedding OR Scalp acupuncture OR Scalp needle OR Scalp electroacupuncture OR scalp stimulation OR Eye needle OR Eye acupuncture OR Abdominal acupuncture OR Abdominal needle OR filiform needle OR silver needle OR three-edged needle OR intradermal needle OR Point application OR needle-embedding OR Catgut Embedding OR pricking therapy OR point injection OR Skin Acupuncture OR transcutanclus electrical acupoint stimulation OR TEAS OR electrical acupoint stimulation OR Acupuncture Point OR Acupoint):ti,ab,kw

#5 #1 OR #2 OR #3 OR #4

#6 MeSH descriptor: [Stroke] explode all trees

#7 (Stroke* OR Apoplexy OR Cerebrovascular Apoplexy OR Apoplexy, Cerebrovascular OR Cerebrovascular Stroke* OR Cerebral Stroke* OR Acute Stroke* OR Embolic Stroke* OR Wake up Stroke* OR Ischemic Stroke* OR Thrombotic Stroke* OR Brain Ischemia OR Stroke*, Cerebrovascular OR Stroke*, Cerebral OR Stroke*, Acute OR Brain Infarction OR Cerebral Infarct* OR Intracerebral Hemorrhage* OR Cerebral Hemorrhage* OR Cerebral Brain Hemorrhage* OR Hemorrhagic stroke* OR Hypertensive Intracerebral Hemorrhage* OR Hemorrhage*, Cerebrum OR Cerebrum Hemorrhage* OR Cerebral Parenchymal Hemorrhage* OR Hemorrhage*, Cerebral Parenchymal OR Parenchymal Hemorrhage*, Cerebral OR Hemorrhage*, Intracerebral OR Hemorrhage*, Cerebral OR Cerebral Hemorrhage* OR Brain Hemorrhage*, Cerebral OR Hemorrhage*, Cerebral Brain OR Cerebrovascular Accident* OR Acute Cerebrovascular Accident* OR CVA* OR Vascular Accident*, Brain OR Brain Vascular Accident*):ti,ab,kw

#8 #6 OR #7

#9 MeSH descriptor: [Dyskinesia] explode all trees

#10 MeSH descriptor: [Hemiplegia] explode all trees

#11 MeSH descriptor: [Paralysis] explode all trees

#12 (Hemiplegia OR Paralysis OR Spasm OR Spasticity OR Dyskinesia OR Monoplegia OR Hemiplegic Gait OR Muscle Hypertonia OR Dystonia OR Muscle Hypotonia OR Paraparesis OR limb function OR Limb Movement OR Limb Impairment OR motor function OR Motor Dysfunction OR Motor Impairment OR Gait Disorder OR Gait Dysfunction OR Limb OR Gait OR motor OR Lower limb OR lower extremities OR upper extremity OR upper limb OR leg OR digit OR toe OR knee OR ankle OR foot OR thigh OR Lower extremity OR shoulder OR arm OR forearm OR wrist OR hand OR Hip joint OR Balance ):ti,ab,kw

#13 #9 OR #10 OR #11 OR #12

#14 #5 AND #8 AND #13

**Embase-790**

**
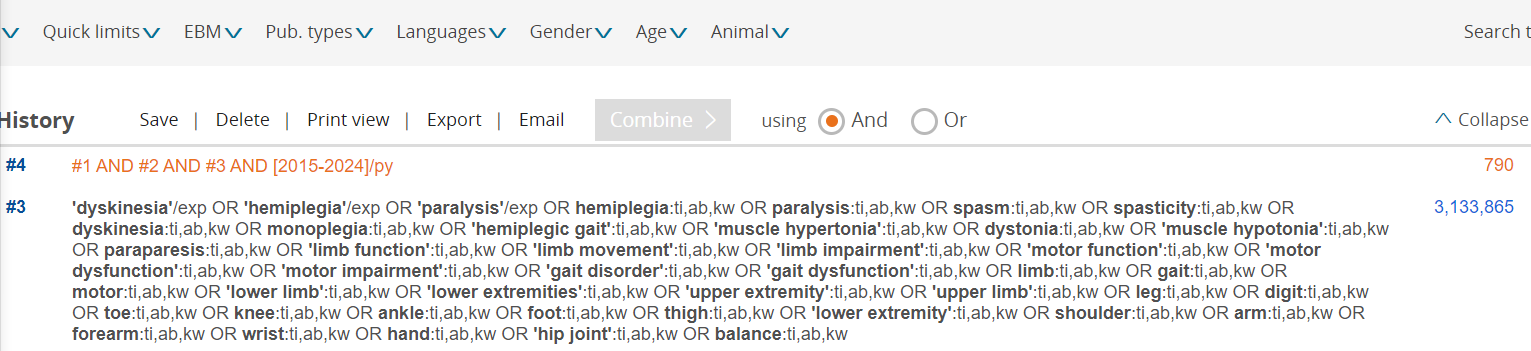
**

#1 'acupuncture'/exp OR 'moxibustion'/exp OR 'acupuncture therapy'/exp OR acupuncture:ti,ab,kw OR 'acupuncture therapy':ti,ab,kw OR 'acupuncture treatment':ti,ab,kw OR 'treatment, acupuncture':ti,ab,kw OR 'therapy, acupuncture':ti,ab,kw OR 'manual acupuncture':ti,ab,kw OR 'acupuncture moxibustion':ti,ab,kw OR moxibustion:ti,ab,kw OR moxabustion:ti,ab,kw OR 'warm needle':ti,ab,kw OR 'warm acupuncture':ti,ab,kw OR thermoacupuncture:ti,ab,kw OR electroacupuncture:ti,ab,kw OR 'electro acupuncture':ti,ab,kw OR 'electric acupuncture':ti,ab,kw OR 'point, acupuncture':ti,ab,kw OR pharmacopuncture:ti,ab,kw OR 'pharmacoacupuncture treatment':ti,ab,kw OR 'treatment, pharmacoacupuncture':ti,ab,kw OR 'pharmacoacupuncture therapy':ti,ab,kw OR 'therapy, pharmacoacupuncture':ti,ab,kw OR 'acupoint injection':ti,ab,kw OR 'acupuncture injection':ti,ab,kw OR 'pharmaco acupuncture':ti,ab,kw OR 'auricular needle':ti,ab,kw OR 'acupunctures, ear':ti,ab,kw OR 'ear acupuncture':ti,ab,kw OR 'ear needle':ti,ab,kw OR 'earlobe acupuncture':ti,ab,kw OR 'auricular acupuncture':ti,ab,kw OR auriculotherapy:ti,ab,kw OR 'acupuncture, auricular':ti,ab,kw OR 'auricular plaster therapy':ti,ab,kw OR 'auricular point sticking':ti,ab,kw OR 'auricular pressure':ti,ab,kw OR 'fire needle':ti,ab,kw OR 'fire acupuncture':ti,ab,kw OR 'acupoint catgut embedding':ti,ab,kw OR 'scalp acupuncture':ti,ab,kw OR 'scalp needle':ti,ab,kw OR 'scalp electroacupuncture':ti,ab,kw OR 'scalp stimulation':ti,ab,kw OR 'eye needle':ti,ab,kw OR 'eye acupuncture':ti,ab,kw OR 'abdominal acupuncture':ti,ab,kw OR 'abdominal needle':ti,ab,kw OR 'filiform needle':ti,ab,kw OR 'silver needle':ti,ab,kw OR 'three-edged needle':ti,ab,kw OR 'intradermal needle':ti,ab,kw OR 'point application':ti,ab,kw OR 'needle embedding':ti,ab,kw OR 'catgut embedding':ti,ab,kw OR 'pricking therapy':ti,ab,kw OR 'point injection':ti,ab,kw OR 'skin acupuncture':ti,ab,kw OR 'transcutanclus electrical acupoint stimulation':ti,ab,kw OR teas:ti,ab,kw OR 'electrical acupoint stimulation':ti,ab,kw OR 'acupuncture point':ti,ab,kw OR acupoint:ti,ab,kw

#2 'stroke'/exp OR 'Apoplexy'/exp OR stroke*:ti,ab,kw OR apoplexy:ti,ab,kw OR 'cerebrovascular apoplexy':ti,ab,kw OR 'apoplexy, cerebrovascular':ti,ab,kw OR 'cerebrovascular stroke*':ti,ab,kw OR 'cerebral stroke*':ti,ab,kw OR 'acute stroke*':ti,ab,kw OR 'embolic stroke*':ti,ab,kw OR 'wake up stroke*':ti,ab,kw OR 'ischemic stroke*':ti,ab,kw OR 'thrombotic stroke*':ti,ab,kw OR 'brain ischemia':ti,ab,kw OR 'stroke*, cerebrovascular':ti,ab,kw OR 'stroke*, cerebral':ti,ab,kw OR 'stroke*, acute':ti,ab,kw OR 'brain infarction':ti,ab,kw OR 'cerebral infarct*':ti,ab,kw OR 'intracerebral hemorrhage*':ti,ab,kw OR 'cerebral brain hemorrhage*':ti,ab,kw OR 'hemorrhagic stroke*':ti,ab,kw OR 'hypertensive intracerebral hemorrhage*':ti,ab,kw OR 'hemorrhage*, cerebrum':ti,ab,kw OR 'cerebrum hemorrhage*':ti,ab,kw OR 'cerebral parenchymal hemorrhage*':ti,ab,kw OR 'hemorrhage*, cerebral parenchymal':ti,ab,kw OR 'parenchymal hemorrhage*, cerebral':ti,ab,kw OR 'hemorrhage*, intracerebral':ti,ab,kw OR 'hemorrhage*, cerebral':ti,ab,kw OR 'cerebral hemorrhage*':ti,ab,kw OR 'brain hemorrhage*, cerebral':ti,ab,kw OR 'hemorrhage*, cerebral brain':ti,ab,kw OR 'cerebrovascular accident*':ti,ab,kw OR 'acute cerebrovascular accident*':ti,ab,kw OR cva*:ti,ab,kw OR 'vascular accident*, brain':ti,ab,kw OR 'brain vascular accident*':ti,ab,kw

#3 'dyskinesia'/exp OR 'hemiplegia'/exp OR 'paralysis'/exp OR hemiplegia:ti,ab,kw OR paralysis:ti,ab,kw OR spasm:ti,ab,kw OR spasticity:ti,ab,kw OR dyskinesia:ti,ab,kw OR monoplegia:ti,ab,kw OR 'hemiplegic gait':ti,ab,kw OR 'muscle hypertonia':ti,ab,kw OR dystonia:ti,ab,kw OR 'muscle hypotonia':ti,ab,kw OR paraparesis:ti,ab,kw OR 'limb function':ti,ab,kw OR 'limb movement':ti,ab,kw OR 'limb impairment':ti,ab,kw OR 'motor function':ti,ab,kw OR 'motor dysfunction':ti,ab,kw OR 'motor impairment':ti,ab,kw OR 'gait disorder':ti,ab,kw OR 'gait dysfunction':ti,ab,kw OR limb:ti,ab,kw OR gait:ti,ab,kw OR motor:ti,ab,kw OR 'lower limb':ti,ab,kw OR 'lower extremities':ti,ab,kw OR 'upper extremity':ti,ab,kw OR 'upper limb':ti,ab,kw OR leg:ti,ab,kw OR digit:ti,ab,kw OR toe:ti,ab,kw OR knee:ti,ab,kw OR ankle:ti,ab,kw OR foot:ti,ab,kw OR thigh:ti,ab,kw OR 'lower extremity':ti,ab,kw OR shoulder:ti,ab,kw OR arm:ti,ab,kw OR forearm:ti,ab,kw OR wrist:ti,ab,kw OR hand:ti,ab,kw OR 'hip joint':ti,ab,kw OR balance:ti,ab,kw

#4 #1 AND #2 AND #3

**CNKI-6660**


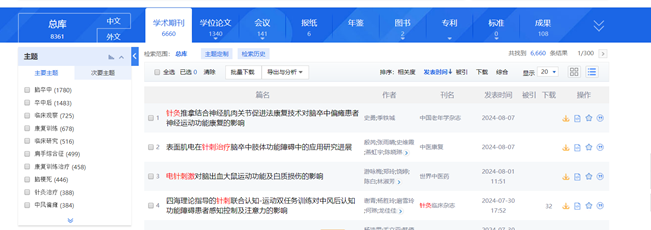


SU%=('针刺'+'电针'+'针灸'+'刺法'+'经皮穴位电刺激'+'隔姜灸'+'隔药灸'+'隔附子饼灸'+'体针'+'耳针'+'头针'+'毫针'+'隔盐灸'+'耳穴贴压'+'热敏灸'+'麦粒灸'+'梅花针'+'穴位敷贴'+'穴位贴敷'+'天灸'+'针刺治疗'+'针灸疗法'+'穴位埋线'+'火针'+'穴位注射'+'艾灸'+'灸法'+'灸疗'+'灸术'+'温针'+'针法'+'三棱针'+'皮肤针'+'芒针'+'眼针'+'手针'+'足针'+'腕踝针'+'平衡针'+'揿针'+'皮内针'+'腹针'+'舌针'+'项针') AND SU%=('中风'+'卒中'+'脑梗死'+'脑梗塞'+'脑出血') AND SU%=('运动障碍'+'运动功能'+'运动功能障碍'+'瘫痪'+'偏瘫' +'半身不遂'+'肢体不利' +'肢体不遂'+'硬瘫'+'软瘫'+'拘挛'+'痉挛'+'迟缓'+'肌张力'+'肌力'+'平衡'+'步行'+'步态' +'共济失调'+'肩手综合征'+'肩关节半脱位'+'肩痛'+'髋踝综合征'+'肢体'+'上肢'+'下肢'+'手' +'腕'+'指'+'足'+'踝'+'膝'+'髋'+'足内翻'+'足外翻'+'足下垂')

**万方-7096**


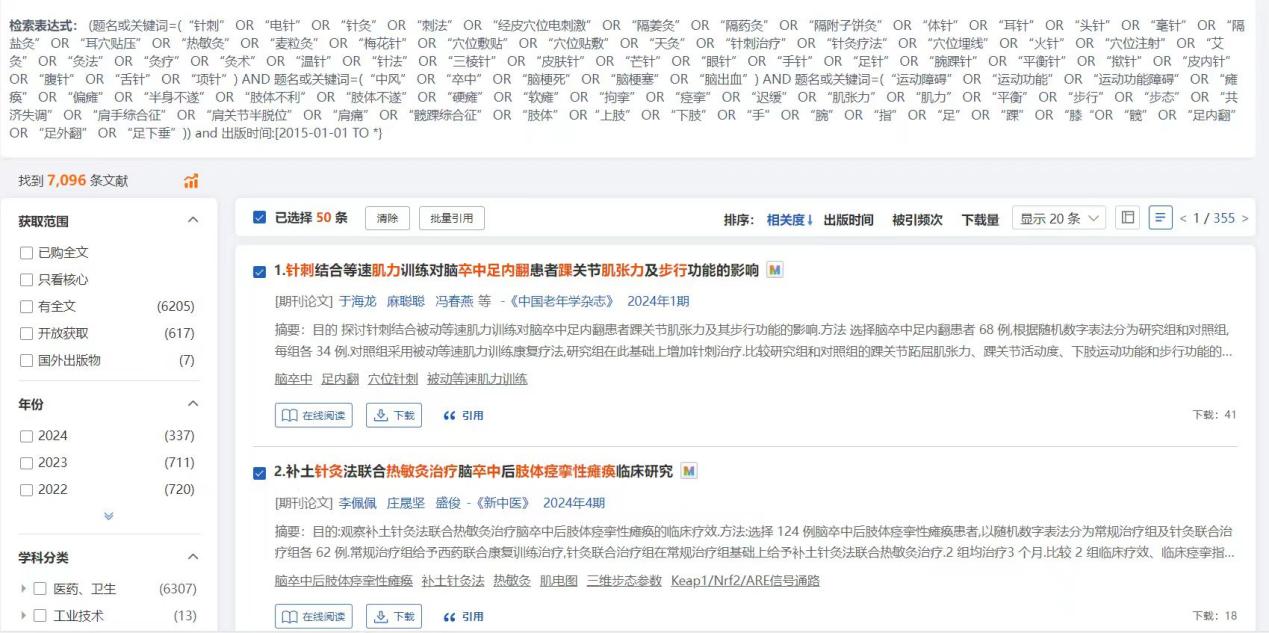


题名或关键词=(“针刺” OR “电针” OR “针灸” OR “刺法” OR “经皮穴位电刺激” OR “隔姜灸” OR “隔药灸” OR “隔附子饼灸” OR “体针” OR “耳针” OR “头针” OR “毫针” OR “隔盐灸” OR “耳穴贴压” OR “热敏灸” OR “麦粒灸” OR “梅花针” OR “穴位敷贴” OR “穴位贴敷” OR “天灸” OR “针刺治疗” OR “针灸疗法” OR “穴位埋线” OR “火针” OR “穴位注射” OR “艾灸” OR “灸法” OR “灸疗” OR “灸术” OR “温针” OR “针法” OR “三棱针” OR “皮肤针” OR “芒针” OR “眼针” OR “手针” OR “足针” OR “腕踝针” OR “平衡针” OR “揿针” OR “皮内针” OR “腹针” OR “舌针” OR “项针”) AND 题名或关键词=(“中风” OR “卒中” OR “脑梗死” OR “脑梗塞” OR “脑出血”) AND 题名或关键词=(“运动障碍” OR “运动功能” OR “运动功能障碍” OR “瘫痪” OR “偏瘫” OR “半身不遂” OR “肢体不利” OR “肢体不遂” OR “硬瘫” OR “软瘫” OR “拘挛” OR “痉挛” OR “迟缓” OR “肌张力” OR “肌力” OR “平衡” OR “步行” OR “步态” OR “共济失调” OR “肩手综合征” OR “肩关节半脱位” OR “肩痛” OR “髋踝综合征” OR “肢体” OR“上肢” OR “下肢” OR “手” OR “腕” OR “指” OR “足” OR “踝” OR “膝“OR “髋” OR “足内翻” OR “足外翻” OR “足下垂”)

**VIP-6006**


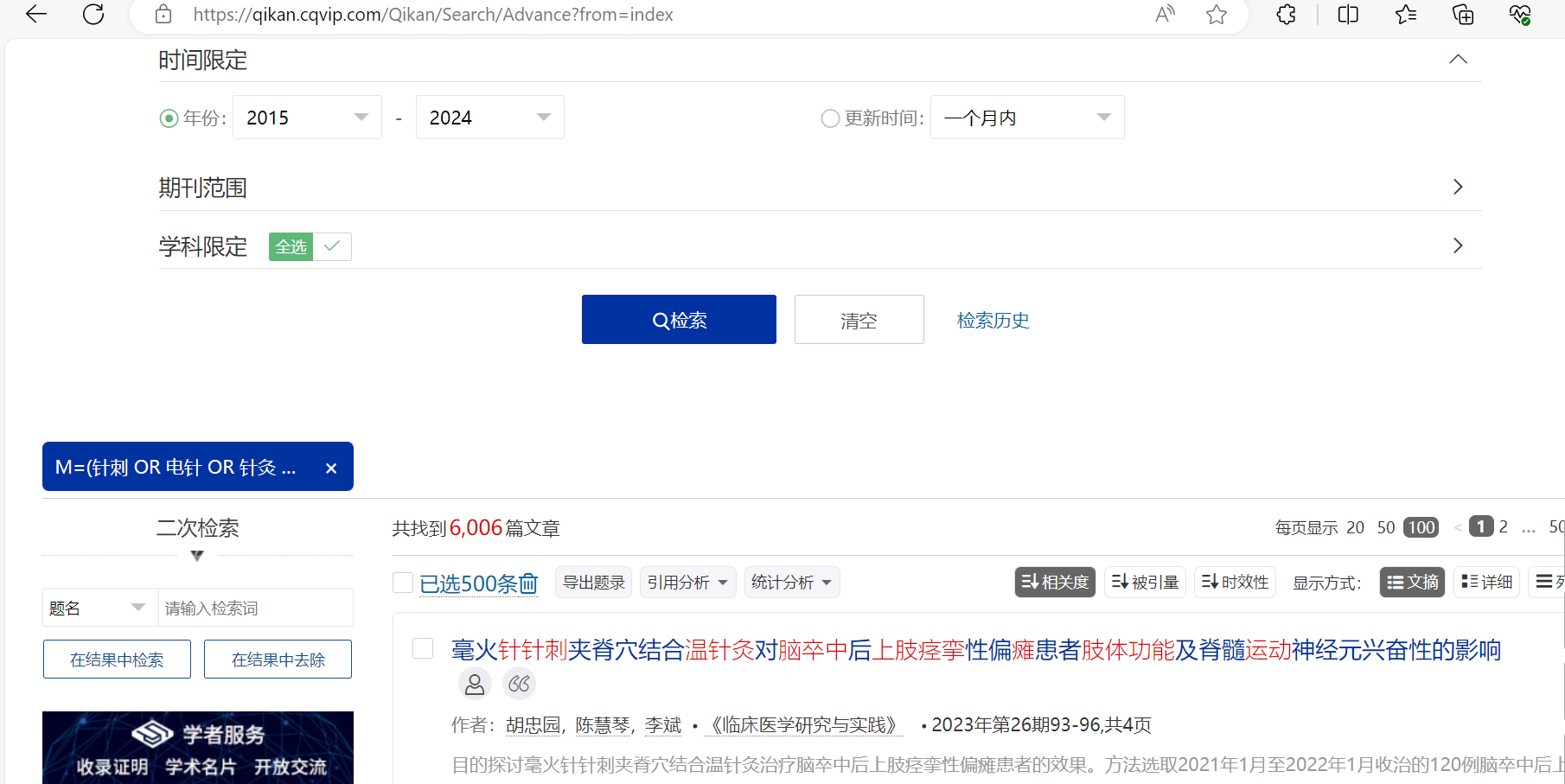


M=(针刺 OR 电针 OR 针灸 OR 刺法 OR 经皮穴位电刺激 OR 隔姜灸 OR 隔药灸 OR 隔附子饼灸 OR 体针 OR 耳针 OR 头针 OR 毫针 OR 隔盐灸 OR 耳穴贴压 OR 热敏灸 OR 梅花针 OR 穴位敷贴 OR 穴位贴敷 OR 天灸 OR 针刺治疗 OR 针灸疗法 OR 穴位埋线 OR 火针 OR 穴位注射 OR 艾灸 OR 灸法 OR 灸疗 OR 灸术 OR 温针 OR 针法 OR 三棱针 OR 皮肤针 OR 芒针 OR 眼针 OR 手针 OR 足针 OR 腕踝针 OR 平衡针 OR 揿针 OR 皮内针 OR 腹针 OR 舌针 OR 项针) AND M=(中风 OR 卒中 OR 脑梗死 OR 脑梗塞 OR 脑出血) AND M=(运动障碍 OR 运动功能 OR 运动功能障碍 OR 瘫痪 OR 偏瘫 OR 半身不遂 OR 肢体不利 OR 肢体不遂 OR 硬瘫 OR 软瘫 OR 拘挛 OR 痉挛 OR 迟缓 OR 肌张力 OR 肌力 OR 平衡 OR 步行 OR 步态 OR 共济失调 OR 肩手综合征 OR 肩关节半脱位 OR 肩痛 OR 髋踝综合征 OR 肢体 OR 上肢 OR 下肢 OR 手 OR 腕 OR 指 OR 足 OR 踝 OR 膝 OR 髋 OR 足内翻 OR 足外翻 OR 足下垂)

**CBM-12469**


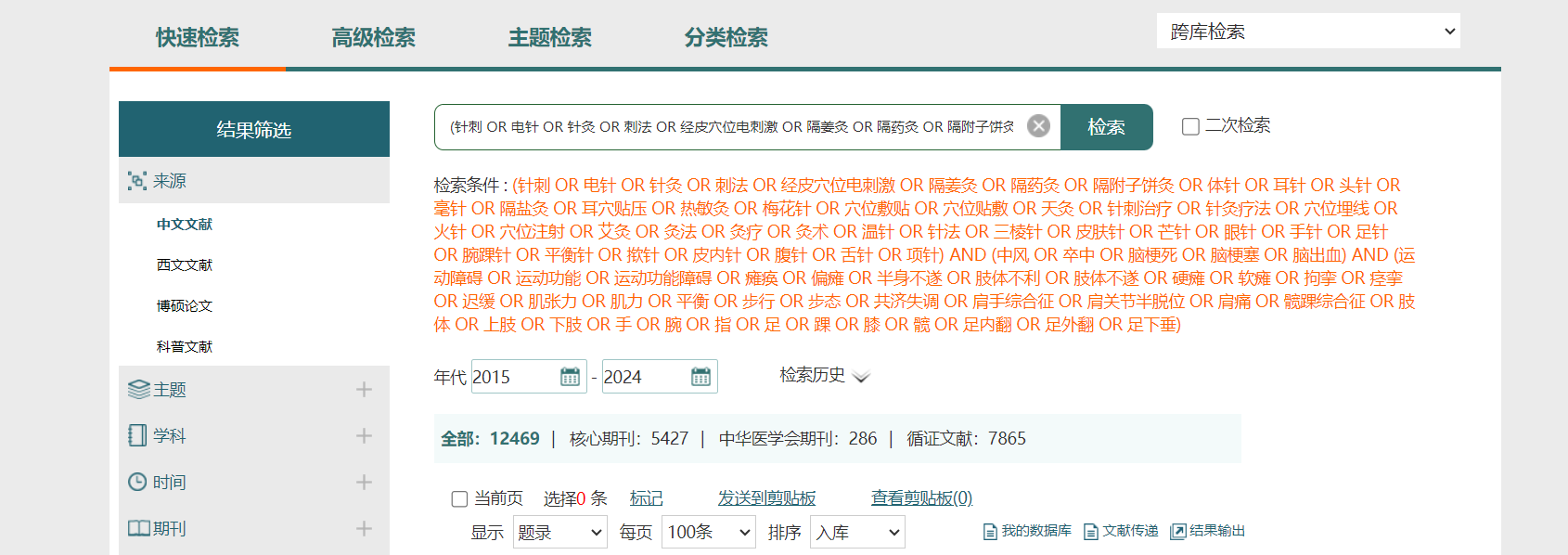


CBM检索式:

【快速检索状态】：

(针刺 OR 电针 OR 针灸 OR 刺法 OR 经皮穴位电刺激 OR 隔姜灸 OR 隔药灸 OR 隔附子饼灸 OR 体针 OR 耳针 OR 头针 OR 毫针 OR 隔盐灸 OR 耳穴贴压 OR 热敏灸 OR 梅花针 OR 穴位敷贴 OR 穴位贴敷 OR 天灸 OR 针刺治疗 OR 针灸疗法 OR 穴位埋线 OR 火针 OR 穴位注射 OR 艾灸 OR 灸法 OR 灸疗 OR 灸术 OR 温针 OR 针法 OR 三棱针 OR 皮肤针 OR 芒针 OR 眼针 OR 手针 OR 足针 OR 腕踝针 OR 平衡针 OR 揿针 OR 皮内针 OR 腹针 OR 舌针 OR 项针) AND (中风 OR 卒中 OR 脑梗死 OR 脑梗塞 OR 脑出血) AND (运动障碍 OR 运动功能 OR 运动功能障碍 OR 瘫痪 OR 偏瘫 OR 半身不遂 OR 肢体不利 OR 肢体不遂 OR 硬瘫 OR 软瘫 OR 拘挛 OR 痉挛 OR 迟缓 OR 肌张力 OR 肌力 OR 平衡 OR 步行 OR 步态 OR 共济失调 OR 肩手综合征 OR 肩关节半脱位 OR 肩痛 OR 髋踝综合征 OR 肢体 OR 上肢 OR 下肢 OR 手 OR 腕 OR 指 OR 足 OR 踝 OR 膝 OR 髋 OR 足内翻 OR 足外翻 OR 足下垂)
