# Supplementary material for: Acupuncture and stroke motor rehabilitation: a decade of evidence synthesis via systematic mapping (2015–2024)
Source: Front Neurol. 2025 Sep 25;16:1647086. doi: 10.3389/fneur.2025.1647086 (PMC12511885; doi:10.3389/fneur.2025.1647086)
Supplement: Supplementary file 3 [file Table_3.DOCX]

| **Supplementary File 3. Intervention and Control groups of the included RCTs** | | | | | | | |
| --- | --- | --- | --- | --- | --- | --- | --- |
| **Design of RCTs** | **Objectives Of trials Design** | **Count** | **Intervention** | **Control** | Control**^＊^** | Control**^＊＊^** | Control**^＊＊＊^** |
| 2-Arm Study | Comparison of different Acupuncture Therapies | 283 | Acup | Acup |  |  |  |
| 2-Arm Study |  | 242 | Acup+RT | Acup+RT |  |  |  |
| 2-Arm Study |  | 165 | M-Acup | Acup |  |  |  |
| 2-Arm Study |  | 34 | M-Acup+RT | M-Acup+RT |  |  |  |
| 2-Arm Study |  | 9 | Acup+CH | Acup+CH |  |  |  |
| 2-Arm Study |  | 5 | Acup+RT+Other | Acup+RT+Other |  |  |  |
| 2-Arm Study |  | 5 | Acup+Other | Acup+Other |  |  |  |
| 2-Arm Study |  | 4 | Acup+RT+CH | Acup+RT+CH |  |  |  |
| 2-Arm Study |  | 1 | Acup+CM | Acup+CM |  |  |  |
| 2-Arm Study |  | 1 | M-Acup+RT+CH | M-Acup+RT+CH |  |  |  |
| 2-Arm Study | Comparison of Compound Effects | 1436 | Acup+RT | RT |  |  |  |
| 2-Arm Study |  | 280 | M-Acup+RT | RT |  |  |  |
| 2-Arm Study |  | 112 | M-Acup+RT | Acup+RT |  |  |  |
| 2-Arm Study |  | 92 | Acup+CH | CH |  |  |  |
| 2-Arm Study |  | 36 | Acup+Other | Other |  |  |  |
| 2-Arm Study |  | 36 | Acup+CM | CM |  |  |  |
| 2-Arm Study |  | 23 | Acup+RT+CH | CH+RT |  |  |  |
| 2-Arm Study |  | 20 | Acup+RT+Other | RT+Other |  |  |  |
| 2-Arm Study |  | 14 | Acup+RT+CM | RT+CM |  |  |  |
| 2-Arm Study |  | 10 | M-Acup+CH | CH |  |  |  |
| 2-Arm Study |  | 8 | Acup+RT | RT+Other |  |  |  |
| 2-Arm Study |  | 6 | M-Acup+Other | Other |  |  |  |
| 2-Arm Study |  | 4 | M-Acup+RT+CH | Acup+RT+CH |  |  |  |
| 2-Arm Study |  | 3 | M-Acup+RT+CM | RT+CM |  |  |  |
| 2-Arm Study |  | 3 | M-Acup+CM | CM |  |  |  |
| 2-Arm Study |  | 2 | M-Acup+RT+CH | CH+RT |  |  |  |
| 2-Arm Study |  | 1 | Acup+CM+Other | CM+Other |  |  |  |
| 2-Arm Study |  | 1 | M-Acup+RT+Other | Acup+RT+Other |  |  |  |
| 2-Arm Study |  | 1 | M-Acup+CH | Acup+CH |  |  |  |
| 2-Arm Study | Comparison of Acupuncture with Other Therapies | 126 | Acup | Blank |  |  |  |
| 2-Arm Study |  | 69 | Acup | RT |  |  |  |
| 2-Arm Study |  | 46 | M-Acup | M-Acup |  |  |  |
| 2-Arm Study |  | 46 | M-Acup | Blank |  |  |  |
| 2-Arm Study |  | 25 | M-Acup | RT |  |  |  |
| 2-Arm Study |  | 21 | Acup | CM |  |  |  |
| 2-Arm Study |  | 15 | Acup | Other |  |  |  |
| 2-Arm Study |  | 11 | Acup+RT | RT+CM |  |  |  |
| 2-Arm Study |  | 7 | M-Acup | CM |  |  |  |
| 2-Arm Study |  | 6 | M-Acup+RT | RT+CM |  |  |  |
| 2-Arm Study |  | 6 | M-Acup | Acup+RT |  |  |  |
| 2-Arm Study |  | 3 | Acup | CH |  |  |  |
| 2-Arm Study |  | 2 | M-Acup | RT+CM |  |  |  |
| 2-Arm Study |  | 2 | M-Acup | CH |  |  |  |
| 2-Arm Study |  | 1 | Acup+Other | CM+Other |  |  |  |
| 2-Arm Study |  | 1 | Acup+CM | RT+CM |  |  |  |
| 2-Arm Study |  | 1 | Acup+CH | CH+RT |  |  |  |
| 2-Arm Study |  | 1 | M-Acup | Acup+Other |  |  |  |
| 2-Arm Study |  | 1 | M-Acup | Acup+CM |  |  |  |
| 2-Arm Study |  | 1 | M-Acup | Other |  |  |  |
| 2-Arm Study | Comparison of Acupuncture Therapy with Sham | 9 | Acup+RT | RT+Sham-Acup |  |  |  |
| 2-Arm Study |  | 7 | Acup | Sham-Acup |  |  |  |
| 2-Arm Study |  | 1 | Acup+RT+Other | Sham-Acup+RT+Other |  |  |  |
| 2-Arm Study |  | 1 | M-Acup | Sham-Acup |  |  |  |
| 3-Arm Study | Comparison of different Acupuncture Therapies | 12 | Acup | Acup | Acup |  |  |
| 3-Arm Study |  | 8 | Acup+RT | Acup+RT | Acup+RT |  |  |
| 3-Arm Study |  | 5 | Acup | Acup | Blank |  |  |
| 3-Arm Study |  | 5 | M-Acup | Acup | Acup |  |  |
| 3-Arm Study |  | 3 | Acup | Acup | RT |  |  |
| 3-Arm Study |  | 3 | M-Acup+RT | M-Acup+RT | M-Acup+RT |  |  |
| 3-Arm Study |  | 3 | M-Acup | Acup | Blank |  |  |
| 3-Arm Study |  | 2 | Acup+RT | Acup+RT | Acup |  |  |
| 3-Arm Study |  | 2 | Acup+RT | Acup+RT | Blank |  |  |
| 3-Arm Study |  | 2 | M-Acup+RT | M-Acup+RT | Acup+RT |  |  |
| 3-Arm Study |  | 2 | M-Acup | M-Acup | Acup |  |  |
| 3-Arm Study |  | 1 | Acup+RT+Other | Acup+RT+Other | Acup |  |  |
| 3-Arm Study |  | 1 | Acup+RT | Acup+RT | RT+Other |  |  |
| 3-Arm Study |  | 1 | Acup+CH | Acup+CH | Blank |  |  |
| 3-Arm Study |  | 1 | Acup | Acup | Other |  |  |
| 3-Arm Study |  | 1 | Acup | Acup | CM |  |  |
| 3-Arm Study |  | 1 | M-Acup+RT+Other | Acup+RT+Other | Acup+RT+Other |  |  |
| 3-Arm Study |  | 1 | M-Acup+Other | M-Acup+Other | Acup |  |  |
| 3-Arm Study |  | 1 | M-Acup | M-Acup | M-Acup |  |  |
| 3-Arm Study |  | 1 | M-Acup | M-Acup | Blank |  |  |
| 3-Arm Study |  | 1 | M-Acup | M-Acup | CM |  |  |
| 3-Arm Study | Others | 4 | Acup+RT+Other | Acup+RT | RT |  |  |
| 3-Arm Study |  | 2 | Acup+RT+CH | Acup+RT | RT |  |  |
| 3-Arm Study |  | 2 | Acup+RT+CH | Acup+RT | RT+CH |  |  |
| 3-Arm Study |  | 2 | M-Acup+CH+CM | M-Acup+CM | CH+CM |  |  |
| 3-Arm Study |  | 1 | Acup+RT+Other | Acup+Other | RT+Other |  |  |
| 3-Arm Study |  | 1 | Acup+RT+CM | Acup+RT | RT |  |  |
| 3-Arm Study |  | 1 | Acup+RT+CM | Acup | RT |  |  |
| 3-Arm Study |  | 1 | Acup+Other | Acup | RT |  |  |
| 3-Arm Study |  | 1 | Acup+CH+RT | Acup+RT | CH+RT |  |  |
| 3-Arm Study |  | 1 | Acup+CH+Other | Acup+Other | Other |  |  |
| 3-Arm Study |  | 1 | Acup+CH | Acup | Blank |  |  |
| 3-Arm Study | Comparison of Compound Effects | 91 | Acup+RT | Acup | RT |  |  |
| 3-Arm Study |  | 51 | Acup+RT | Acup+RT | RT |  |  |
| 3-Arm Study |  | 16 | Acup+RT+Other | Acup+RT | RT+Other |  |  |
| 3-Arm Study |  | 11 | Acup+RT | RT | RT |  |  |
| 3-Arm Study |  | 11 | Acup+Other | Acup | Other |  |  |
| 3-Arm Study |  | 11 | Acup+CH | CH | Acup |  |  |
| 3-Arm Study |  | 10 | M-Acup+RT | Acup+RT | RT |  |  |
| 3-Arm Study |  | 9 | M-Acup+RT | RT | M-Acup |  |  |
| 3-Arm Study |  | 5 | Acup+RT+Other | RT+Other | RT |  |  |
| 3-Arm Study |  | 5 | M-Acup+RT | Acup+RT | Acup+RT |  |  |
| 3-Arm Study |  | 5 | M-Acup+RT | M-Acup+RT | RT |  |  |
| 3-Arm Study |  | 4 | Acup+RT | RT | Blank |  |  |
| 3-Arm Study |  | 4 | M-Acup+RT | M-Acup | RT |  |  |
| 3-Arm Study |  | 3 | M-Acup+RT | Acup+RT | M-Acup |  |  |
| 3-Arm Study |  | 2 | M-Acup+RT | RT | Acup |  |  |
| 3-Arm Study |  | 1 | Acup+RT+Other | RT+Other | Acup |  |  |
| 3-Arm Study |  | 1 | Acup+RT+Other | RT+Other | Blank |  |  |
| 3-Arm Study |  | 1 | Acup+RT+CH | CH+RT | RT |  |  |
| 3-Arm Study |  | 1 | Acup+RT+CH | CH+RT | Blank |  |  |
| 3-Arm Study |  | 1 | Acup+RT | RT | CM |  |  |
| 3-Arm Study |  | 1 | Acup+Other | RT | Other |  |  |
| 3-Arm Study |  | 1 | Acup+CM+RT | CM+RT | CM |  |  |
| 3-Arm Study |  | 1 | Acup+CM | Acup+RT | RT+CM |  |  |
| 3-Arm Study |  | 1 | Acup+CH+RT | CH+RT | Blank |  |  |
| 3-Arm Study |  | 1 | Acup+CH+Other | Acup | CH |  |  |
| 3-Arm Study |  | 1 | Acup+CH | Acup+CH | CH |  |  |
| 3-Arm Study |  | 1 | Acup+CH | CH | Blank |  |  |
| 3-Arm Study |  | 1 | M-Acup+RT+Other | RT+Other | Acup |  |  |
| 3-Arm Study |  | 1 | M-Acup+RT+Other | RT+Other | RT |  |  |
| 3-Arm Study |  | 1 | M-Acup+RT | RT | RT |  |  |
| 3-Arm Study |  | 1 | M-Acup+RT | RT | Blank |  |  |
| 3-Arm Study |  | 1 | M-Acup+Other | M-Acup | Other |  |  |
| 3-Arm Study | Comparison of Acupuncture with Other Therapies | 3 | Acup+RT | CM+RT | RT |  |  |
| 3-Arm Study |  | 2 | Acup+RT | RT+Other | RT |  |  |
| 3-Arm Study |  | 1 | Acup+RT | RT+Other | Acup |  |  |
| 3-Arm Study |  | 1 | Acup+RT | CM+RT | Acup |  |  |
| 3-Arm Study |  | 1 | Acup | Other | Other |  |  |
| 3-Arm Study |  | 1 | M-Acup+RT | RT+Other | RT |  |  |
| 3-Arm Study |  | 1 | M-Acup+RT | CH+RT | RT |  |  |
| 3-Arm Study | Comparison of Acupuncture Therapy with Sham | 1 | Acup+RT+Other | RT+Sham-Acup+Other | RT+Acup+Sham-Other |  |  |
| 3-Arm Study |  | 1 | Acup+RT | Sham-Acup+RT | Acup+RT |  |  |
| 3-Arm Study |  | 1 | Acup | Acup | Sham-Acup |  |  |
| 3-Arm Study |  | 1 | Acup | Sham-Acup | Blank |  |  |
| 3-Arm Study |  | 1 | M-Acup+RT | RT+Acup+Sham-Acup | RT |  |  |
| 4-Arm Study | Comparison of different Acupuncture Therapies | 8 | Acup | Acup | Acup | Acup |  |
| 4-Arm Study |  | 5 | Acup+RT | Acup+RT | Acup+RT | RT |  |
| 4-Arm Study |  | 4 | Acup+RT | Acup+RT | RT | RT |  |
| 4-Arm Study |  | 3 | Acup+RT | Acup+RT | Acup+RT | Acup+RT |  |
| 4-Arm Study |  | 2 | M-Acup | M-Acup | M-Acup | M-Acup |  |
| 4-Arm Study |  | 1 | Acup+RT | Acup+RT | RT+Sham-Acup | Acup+RT |  |
| 4-Arm Study |  | 1 | Acup+Other | Acup+Other | Other | Acup |  |
| 4-Arm Study |  | 1 | M-Acup+RT | M-Acup+RT | M-Acup+RT | M-Acup+RT |  |
| 4-Arm Study |  | 1 | M-Acup | Acup | Acup | Acup |  |
| 4-Arm Study | Others | 7 | Acup+RT+Other | Acup+RT | RT+Other | RT |  |
| 4-Arm Study |  | 1 | Acup+RT+CH | Acup+RT | CH+RT | RT |  |
| 4-Arm Study |  | 1 | Acup+RT+CH | M-Acup+RT | CH+RT | RT |  |
| 4-Arm Study |  | 1 | Acup+CH+RT | Acup+RT | CH+RT | RT |  |
| 4-Arm Study |  | 1 | Acup+CH | RT | Acup | CH |  |
| 4-Arm Study | Comparison of Compound Effects | 4 | Acup+RT | Acup | RT | Blank |  |
| 4-Arm Study |  | 4 | Acup+CH | Acup | CH | Blank |  |
| 4-Arm Study |  | 3 | Acup+Other | Acup | Other | Blank |  |
| 4-Arm Study |  | 1 | Acup+RT | Acup | RT | RT |  |
| 4-Arm Study |  | 1 | Acup+Other | Other | RT | Acup |  |
| 4-Arm Study |  | 1 | M-Acup+RT+Other | Acup+RT+Other | M-Acup+RT | Acup+RT |  |
| 4-Arm Study |  | 1 | M-Acup+RT | Acup+RT | M-Acup | Acup |  |
| 4-Arm Study |  | 1 | M-Acup+RT | M-Acup | M-Acup | RT |  |
| 4-Arm Study |  | 1 | M-Acup+RT | M-Acup | RT | Blank |  |
| 5-Arm Study | Comparison of different Acupuncture Therapies | 1 | M-Acup | Acup | M-Acup | M-Acup | M-Acup |

**Abbreviations:** Acup: Acupuncture; M-Acup: Multi-Acupuncture; RT: Rehabilitation Therapy；CH: Chinese Herb; CM: Conventional Medicine.
