# Supplementary material for: Acupuncture and stroke motor rehabilitation: a decade of evidence synthesis via systematic mapping (2015–2024)
Source: Front Neurol. 2025 Sep 25;16:1647086. doi: 10.3389/fneur.2025.1647086 (PMC12511885; doi:10.3389/fneur.2025.1647086)
Supplement: Supplementary file 4 [file Table_4.DOCX]

**Supplementary File 4. Outcome characteristics of the included RCTs**

| **Outcomes** | **N** |
| --- | --- |
| **1 Clinical Outcome Assessment Scales of Motor Function** | 6117 |
| **Neurological deficit scores(927):** NIHSS(591);uncertain(103);NDS(93);CSS1(52);mRS(27);NFDS(13);MESSS(11);SSS(11);CCS(8);CNS(4);ESS(4);CNDS(2);CNFDS(2);GOS(2);NBD(2);NDF(2) motor function(2152)： FMA(2037); uncertain(80); FMMS(14); MSS(5); SFMA(4); GMFM(4); RMI(2); STREAM(2); COPM(1); JOA(1); MAL(1); MCT(1) Upper limb: FMA-UE(307)；WMFT(42); DASH(16); STEF(11); UEFT(10); The Ueda Senshiro Hemiplegic Function Evaluation Method(4); FTHUE-HK(1); MI(1) Shoulder/Hand:： SHSS(45); ARAT(19); FMA-F(15); finger function-uncertain(23); CMS(8); Lindmark Wrist motor function score(11); Broetz scale Hand Function Scale (3); Carrol Hand function evaluation scale(3); JTHFT(3); SPADI(2); Melle grade(1); High Bank Shoulder Functional Assessment Scale (Pain part) (1); Shoulder function Scale (C-M) score(1); The US Miclael Reese score(1); UCLA(1); ASES(1); Task specific test-he Purdue pegboard test was used to determine dexterity(1); MAL-AOU(1); Finger Tapping Test(1) Lower limbs/foot： FMA-L(152); AOFAS(3); Chedoke-Mc Master Stroke evaluation form(1); Maryland Foot motor function scoring criteria(1); Vusl Mever Lower extremity motor score (1); IRISSG (1); (MI-L)(1) Balance, trunk, movement, standing and extremity postures. BBS(340); FMA-B(42); Sheikh Tso Control Measurement Scale (15); ICARS(14); PASS(12); TCT(9); TIS(5); BBA(4); BLS(3); mini BESTest(3); Three-level balance determination (2); Romberg Rate Simple balance function determination (1); SCP(1); Evaluation of non-balanced coordination test (1); evaluation of balanced coordination test (1); CRT(1); PST(1); Self-proposed ataxia scale (1); station transfer capacity (1); EMS(1); MRMI(1); POMA(3) Walk and gait HFAC(98); FAC(73); TUG(36); TGA(24); FGA(5); Hoffer Classification of walking ability(3); WGS (4); Parker-Palmer Walking Interaction Strength Scale (1); Walking ability score -uncertain(4); Carr-shepherd Walking Rating Scale (2) Spasticity / muscle tone: MAS(776); CSI(162); Brunnstrom Stage evaluation (114); CSS2(36); MTS(4); Penn Score (2); Muscle tone score(10) pain: VAS(399); McGill Pain Scale (28); NRS(21); Pain score-uncertain (15); NPRS(3); Wong Baker facial expression scale(2); BRS-6(1); Fugl Meyer Pain Assessment(1); BPI(1); PRI(1); VRS(1); FIQ(1); Facial expression Rating Scale-Based Digital Rating Scale (NRS-FRS) (2) Sensation: FMA-S(5); Lindmark Sensory Function Scale Scale (1); SFS Scale (1); reNSA (1); SIS (1) other: Improvement in clinical symptoms / points (41); BLS score(1); ICARS Surgical Recovery Scale (1)；Bobath Index(1) |  |
| **2 Scales of Accompanying Symptoms** | 129 |
| **Emotional / Psychological status:** SAS(17); SDS(15); HAMD(13); HAMA(8); BAI(2); SCL-90(2); BDI(1); HADS(1); ABC(1); WEM WBS(1); STAI(1); DASS-21(1); mental status-uncertain(1) swallowing function: Wada water drinking test(3);GUSS(1); BSA(1); Metrohealh Swallowing function score (1); EAT-10(1); VFSS(2); Water intake test score (1); SSA(1); Burke grade(1) Cognition function: MMSE(24); MoCA(11); LOTCA(1); CDR(1); HAMD(1); WAIS-III(1); HDS(1); Cognitive ability score-uncertain(1) Language function: Language function score-uncertain(3); Aphasia Battery of Chinese(2); BDAE(2) other: PSQI(5); Modified Portmann scale score(1) |  |
| **3 Scales of Life Quanlity** | 2629 |
| BI(896); MBI(755); ADL(380); SF-36(137); SS-QOL(119); activity of daily living -uncertain(55); FIM(54); FCA(52); Quality of life -uncertain (52); WHO-QOL/WHO-QOL-BREF(45); GQOLI-74(19); GQOL-74(16); QOL/QOL-BREF(16); SIS(11); QLQ-C30(6); KPS Quality of Life Scale (2); FAQ(2); QLI(2); Comprehensive Functional Score (2); PULSES Rating Scale (1); simple health scale (1); TAA(1); BBSI(1); Behavior ability score (1); self-efficacy score (1) |  |
| **4 TCM Syndrome Scores** | 211 |
| **5 Objective Examination of Motor Function** | 1598 |
| Laboratory examination(358)；ROM(210); walking gait examination(187); sEMG examination(170); muscle strength examination(165); edema/swelling/circumdiameter measurement (127); ultrasound examination(103)；electrophysiological examination(74)；Imaging examination(59); dorsal flexion, plantar flexion, valgus, and varus degree(49); Balance examination(35); isokinetic muscle strength examination(13); EEG examination（6）；handgrip strength examination(6)；Knee hyperextension / knee distraction assessment (5); others(31) |  |
| **6 Effective Rate of Treatment** | 2047 |
| **7 Safety and adverse effects** | 330 |
| **8 Health economic indicators** | 21 |
| **9 Patient expectations and satisfaction** | 58 |
| **10 Others** | 77 |
| Overall complication rate (23);Disability / death (10); PRO(10);SIAS(9); Cooperativeness (7);OHS(4); GEPI(2); other(12) |  |

**Abbreviations of outcomes:**

NIHSS: National Institutes of Health Stroke Scale; NDS: Nerve Deficiency Scale;The CSS-1: Chinese Stroke Scale; mRS: modified Rankin scale; NFDS: Neural function defect score; MESSS: Modified Edinburgh‐Scandinavian Stroke Scale; SSS: Scandinavian Stroke Scale; CCS: China clinical neurological deficit in stroke scale; ESS: European Stroke Scale; CNS: clinicalnervefunctionIim-itationscores; GOS: Glasgow Outcome Scale; CNDS: Clinical Neurological Disfunction Scale; CNFDS: Clinical neural function defect scale; NDF: The degree of neurological deficit score standard disability grade score; NBD: neurologicadeficit score; FMA: Fugl-Meyer Motor Assessment; FMMS: Fugl-Meyer assessment scale; SFMA: Selective Functional Movement Assessment; RMI: Rivermead Movement index; CMFM: Crude Motor Function Scale; STREAM: the Stroke Rehabilitation Assessment of Movement; MSS, motor status score; COPM: Canadian Occupational Performance Measure; JOA: Japanese Orthopaedic Association Assessment Treatment Score; MAL: Motor Activity Log; MCT: Motor Control Test; FMA-UE: Fugl-Meyer assessment for the upper extremity; WMFT: Wolf Motor Function Test; DASH: DASH scale; STEF: Simple Test for Evaluating Hand Function; UEFT: Upper Extremity Function Test; The Ueda Senshiro Hemiplegic Function Evaluation Method; FTHUE-HK: the score of hemiplegic upper limb function test (Hong Kong version); MI: Upper extremity Motor Force Index (MI); SHSS: shoulder-hand syndrome scale; ARAT: Action Research Arm Test;FMA-F:Fugl-meyer assessment of finger; CMS: Constant-Murley Shoulder Function Scale; JTHFT: Jebsen Taylor Hand function test; SPADI: Shoulder Pain and Disability Index; UCLA: University of California Shoulder Score; ASES: American Association of Shoulder and Elbow Surgery score; MAL-AOU: Motor Activity Log Amount of Use; FMA‐LE, Fugl–Meyer assessment of lower extremity subscore; AOFAS: ankle-hind foot function evaluation; IRISSG: International Restless Leg Syndrome Study Group Classification; MI-L: lower extremity muscle strength of Motricity index; BBS: Berg Balance Scale; FMA-B: The Fugl-Meyer Assessment Scale of Balance Functional; ICARS: International Cooperative Ataxia Rating Scale; PASS:  Postural Assessment Scale; TCT: Trunk Control Test; TIS: Trunk Dysfunction Scale; BBA: Brunel Balanced Assessment; BLS: Burke Lateropulsion Scale; mini BESTest: Mini Balance Evaluation Systems Test; SCP: Scale for Contraversive Pushing; MCT: Motor Control Test; CRT: Stand-up tes; PST: Postural stability test; EMS: Elderly Mobility Scale; MRMI: United Kingdom Medical Research Council MRMI Scale; POMA: Performance-Oriented Mobility Assessment scale; HFAC: Holden Functional Ambulation Category; FAC: Functional Ambulation Category; TUG: The Timed Up & Go; TGA: Tinetti Gait Assessment Scale; FGA: Functional Gait Evaluation Scale; WGS: Wisconsin Gait Scale; MAS：Modified Ashworth Scale； CSI: composite spasticity index；CSS：composite spasticity score；MTS: Modified Tardieu Scale

VAS: Visual Analog Scale/Score; NRS: Numeric Rating Scale; NPRS: Numerical Pain Rating Index Scale; BRS: Behavioral Pain Scale; BPI: Brief Pain Inventory; PRI: Pain rating index; VRS: visual rating scale Intensities of pain; FIQ: Fibromyalgia Impact Questionnaire; NRS-FRS: Facial expression Rating Scale-Based Digital Rating Scale; FMA-S: Fugl-Meyer Scale of SensFunction; SFS: Sensory Function Assessment; reNSA: revised Nottingham Sensory Assessment Scale; SIS: Sensory Index Scale; BI: Barthel Index; MBI: Modified Barthel Index; ADL: activities of daily living; SF-36: Quality of Life-36; SS-QOL: Stroke-Specific Quality of Life; FIM: functional independence measure; FCA: Functional Capacity Assessment; WHO-QOL-BREF: World Health Organization Quality of Life Brief Scale; WHO-QOL: World Health Organization Quality of Life Scale ; GQOLI-74: General Quality of Life Unventory-74; GQOL-74: Generic quality of life inventory-74; SIS: stroke impact scale; QLQ-C30: European Organisation for Research and Treatment of Cancer Quality of Life Questionnaire C30; FAQ :  functional activities questionnaire; QLI: Quality of Life Index; TAA: Task Analysis Assessment, BBSI: Behavioral Based Safety Index; SAS: Self-rating Anxiety Scale score; SDS:self-rating depression scale; HAMD: Hamilton Depression Rating Scale; HAMA: Hamilton Anxiety Scale; BAI: Beck Anxiety Inventory; SCL-90: Subscale of Symptom-Checklist; BDI: Beck Depression Inventory; ABC: Activities-specific Balance Confidence; WEMWBS: Warwick-Edinburgh Mental Well-being Scale; DASS-21: Depression, Anxiety and Stress Scales; HAD: Hospital Anxiety and Depression Scale; STAI: State-Trait Anxiety Inventory; GUSS: Gugging Swallowing Function Assessment Scale; BSA: Based on the bedside swallowing assessment; EAT-10: Eating Assessment Tool-10; VFSS: Videofluoroscopic Swallowing Study; SSA: Standardized Swallowing Assessment; MMSE: mini-mental state examination; MoCA: Montreal Cognitive Assessment scale; LOTCA: Loewenstein Occupational Therapy Cognitive Assessment; CDR: Clinical Dementia Rating Scale; HAMD: Hamilton Depression Scale; WAIS-III: Wechsler Adult Intelligence Scale, third edition; HDS: Hasegawa dementia scale; BDAE: The semantic score of the Boston Diagnostic Aphasia Examination; PSQI : Pittsburgh Sleep Quality Index; PRO: Patient-Reported Outcome Measures; SIAS: Stroke Impairment Assessment Set; OHS: Oxford Handicap Score; GEPI: GuidestotheEvaluationofPermanentImpairment
