# Supplementary material for: Acupuncture and stroke motor rehabilitation: a decade of evidence synthesis via systematic mapping (2015–2024)
Source: Front Neurol. 2025 Sep 25;16:1647086. doi: 10.3389/fneur.2025.1647086 (PMC12511885; doi:10.3389/fneur.2025.1647086)
Supplement: Supplementary file 5 [file Table_5.DOCX]

**Supplementary File 5. Statistical analysis of the frequency of acupoint related information in post-stroke motor impairment.**

| **Sort** | **Acupoints** | **Count** | **Proportion** |
| --- | --- | --- | --- |
| 1 | LI4 | 2321 | 5.5% |
| 2 | LI11 | 2274 | 5.4% |
| 3 | ST36 | 1938 | 4.6% |
| 4 | LI15 | 1775 | 4.2% |
| 5 | SJ5 | 1761 | 4.2% |
| 6 | SP6 | 1616 | 3.8% |
| 7 | GB34 | 1595 | 3.8% |
| 8 | LI10 | 1587 | 3.8% |
| 9 | PC6 | 1235 | 2.9% |
| 10 | LR3 | 997 | 2.4% |
| 11 | LU5 | 894 | 2.1% |
| 12 | GB30 | 831 | 2.0% |
| 13 | DU20 | 823 | 2.0% |
| 14 | HT1 | 709 | 1.7% |
| 15 | BL40 | 709 | 1.7% |
| 16 | DU26 | 656 | 1.6% |
| 17 | GB39 | 654 | 1.6% |
| 18 | SP10 | 644 | 1.5% |
| 19 | ST41 | 639 | 1.5% |
| 20 | SJ14 | 528 | 1.3% |

| **Hemiplegia** | | | | | **spastic and paralysis** | | | | |
| --- | --- | --- | --- | --- | --- | --- | --- | --- | --- |
| **Sort** | **Acupoints** | **frequency** | **High-frequency Link** | **frequency** | **Sort** | **Acupoints** | **frequency** | **High-frequency Link** | **frequency** |
| 1 | Hegu(LI4) | 1559 | LI4 - LI11 | 1249 | 1 | Hegu(LI4) | 88 | LI4 - LI11 | 67 |
| 2 | Quchi(LI11) | 1530 | LI11 - ST36 | 1193 | 2 | Quchi(LI11) | 74 | LI4 - LI15 | 59 |
| 3 | Zusanli(ST36) | 1506 | LI4 - ST36 | 1169 | 3 | Waiguan(SJ5) | 73 | LI15 - LI11 | 56 |
| 4 | Sanyinjiao(SP6) | 1261 | LI4 - GB34 | 987 | 4 | Jianyu(LI15) | 66 | LI4 - SJ5 | 55 |
| 5 | Yanglingquan(GB34) | 1247 | LI11 - GB34 | 953 | 5 | Sanyinjiao(SP6) | 60 | LI11 - SJ5 | 52 |
| 6 | Waiguan(SJ5) | 1096 | ST36 - GB34 | 923 | 6 | Shousanli(LI10) | 59 | LI15 - SJ5 | 49 |
| 7 | Shousanli(LI10) | 1061 | LI11 - SJ5 | 914 | 7 | Zusanli(ST36) | 57 | LI4 - ST36 | 48 |
| 8 | Jianyu(LI15) | 1039 | LI4 - SJ5 | 906 | 8 | Yanglingquan(GB34) | 56 | LI4 - GB34 | 47 |
| 9 | Neiguan(PC6) | 913 | LI4 - LI10 | 891 | 9 | Neiguan(PC6) | 51 | LI10 - SJ5 | 47 |
| 10 | Taichong(LR3) | 781 | LI4 - LI15 | 866 | 10 | Chize(LU5) | 43 | LI4 - LI10 | 45 |
| 11 | Huantiao(GB30) | 710 | LI11 - LI10 | 853 | 11 | Weizhong(BL40) | 30 | LI15 - GB34 | 44 |
| 12 | Baihui(DU20) | 661 | LI11 - LI15 | 835 | 12 | Huantiao(GB30) | 29 | LI11 - ST36 | 44 |
| 13 | Chize(LU5) | 639 | ST36 - SJ5 | 822 | 13 | Jiquan(HT1) | 28 | LI4 - SP6 | 42 |
| 14 | Weizhong(BL40) | 563 | ST36 - SP6 | 814 | 14 | Taichong(LR3) | 28 | LI11 - LI10 | 41 |
| 15 | Xuehai(SP10) | 529 | ST36 - LI10 | 810 | 15 | Yinlingquan(SP9) | 28 | LI11 - GB34 | 41 |
| 16 | Shuigou(DU26) | 525 | LI4 - SP6 | 804 | 16 | Baihui(DU20) | 27 | GB34 - ST36 | 40 |
| 17 | Jiquan(HT1) | 515 | ST36 - LI15 | 758 | 17 | Tianjing(SJ10) | 21 | LI15 - LI10 | 38 |
| 18 | Xuanzhong(GB39) | 463 | LI11 - SP6 | 758 | 18 | Houxi(SI3) | 20 | LI15 - ST36 | 38 |
| 19 | Jiexi(ST41) | 452 | GB34 - LI10 | 729 | 19 | Binao(LI14) | 19 | SP6 - ST36 | 38 |
| 20 | Fengshi(GB31) | 417 | GB34 - SJ5 | 728 | 20 | Kunlun(BL60) | 19 | PC6 - SP6 | 38 |

| **Upper limb** | | | | | **Lower limb** | | | | |
| --- | --- | --- | --- | --- | --- | --- | --- | --- | --- |
| **Sort** | **Acupoints** | **frequency** | **High-frequency Link** | **frequency** | **Sort** | **Acupoints** | **frequency** | **High-frequency Link** | **frequency** |
| 1 | Jianyu(LI15) | 663 | LI11 - SJ5 | 508 | 1 | Zusanli(ST36) | 277 | GB34 - ST36 | 178 |
| 2 | Hegu(LI4) | 655 | LI4 - SJ5 | 492 | 2 | Yanglingquan(GB34) | 261 | LR3 - ST36 | 125 |
| 3 | Quchi(LI11) | 646 | LI11 - LI4 | 458 | 3 | Sanyinjiao(SP6) | 190 | SP6 - ST36 | 120 |
| 4 | Waiguan(SJ5) | 581 | SJ5 - LI10 | 450 | 4 | Taichong(LR3) | 150 | GB39 - GB34 | 116 |
| 5 | Shousanli(LI10) | 455 | LI4 - LI10 | 427 | 5 | Jiexi(ST41) | 148 | ST41 - ST36 | 111 |
| 6 | Jianliao(SJ14) | 345 | LI11 - LI10 | 421 | 6 | Xuanzhong(GB39) | 140 | ST41 - GB34 | 107 |
| 7 | Jianzhen(SI9) | 273 | SJ5 - SJ14 | 358 | 7 | Hegu(LI4) | 109 | LR3 - GB34 | 105 |
| 8 | Neiguan(PC6) | 258 | LI11 - SJ14 | 340 | 8 | Qiuxu(GB40) | 108 | SP6 - GB34 | 99 |
| 9 | Binao(LI14) | 241 | LI4 - SJ14 | 338 | 9 | Quchi(LI11) | 98 | GB39 - ST36 | 91 |
| 10 | Chize(LU5) | 210 | SJ14 - LI10 | 335 | 10 | Xuehai(SP10) | 89 | LI4 - ST36 | 84 |
| 11 | Houxi(SI3) | 173 | SI9 - LI4 | 308 | 11 | Yinlingquan(SP9) | 86 | LI4 - LI11 | 83 |
| 12 | Sanyinjiao(SP6) | 165 | SI9 - SJ5 | 238 | 12 | Waiguan(SJ5) | 84 | GB40 - GB34 | 83 |
| 13 | Zusanli(ST36) | 160 | LI4 - PC6 | 235 | 13 | Zhaohai(KI6) | 84 | SP6 - LR3 | 76 |
| 14 | Jiquan(HT1) | 156 | SI9 - LI10 | 216 | 14 | Weizhong(BL40) | 78 | LI4 - GB34 | 73 |
| 15 | Jianqian(EX-UE12) | 137 | LI11 - SI9 | 214 | 15 | Huantiao(GB30) | 76 | LI11 - SJ5 | 71 |
| 16 | Jianjing(GB21) | 122 | LI14 - LI4 | 211 | 16 | Jianyu(LI15) | 73 | LI11 - ST36 | 71 |
| 17 | Tianzong(SI11) | 116 | PC6 - SJ5 | 182 | 17 | Shousanli(LI10) | 72 | ST41 - GB39 | 70 |
| 18 | Ashi point(Ashi point) | 100 | SI9 - PC6 | 181 | 18 | Baihui(DU20) | 67 | LI4 - SJ5 | 67 |
| 19 | Baihui(DU20) | 98 | LI14 - SJ5 | 169 | 19 | Anterior parietal temporal oblique line(MS6) | 66 | SP6 - SP9 | 66 |
| 20 | Baxie(EX-UE9) | 94 | LI14 - LI11 | 164 | 20 | Neiguan(PC6) | 64 | LI11 - GB34 | 65 |

| **spastic paralysis** | | | | | **flaccid paralysis** | | | | |
| --- | --- | --- | --- | --- | --- | --- | --- | --- | --- |
| **Sort** | **Acupoints** | **frequency** | **High-frequency Link** | **frequency** | **Sort** | **Acupoints** | **frequency** | **High-frequency Link** | **frequency** |
| 1 | Hegu(LI4) | 614 | LI4 - LI11 | 468 | 1 | Quchi(LI11) | 73 | LI4 - LI11 | 61 |
| 2 | Quchi(LI11) | 580 | LI11 - SJ5 | 393 | 2 | Hegu(LI4) | 72 | LI11 - ST36 | 55 |
| 3 | Waiguan(SJ5) | 494 | LI4 - SJ5 | 375 | 3 | Zusanli(ST36) | 63 | LI15 - LI11 | 49 |
| 4 | Yanglingquan(GB34) | 480 | LI4 - LI10 | 363 | 4 | Jianyu(LI15) | 58 | LI4 - ST36 | 48 |
| 5 | Zusanli(ST36) | 455 | LI15 - LI11 | 351 | 5 | Waiguan(SJ5) | 52 | LI4 - LI15 | 46 |
| 6 | Jianyu(LI15) | 450 | LI11 - LI10 | 348 | 6 | Sanyinjiao(SP6) | 50 | LI4 - SJ5 | 43 |
| 7 | Shousanli(LI10) | 440 | LI4 - LI15 | 346 | 7 | Taichong(LR3) | 49 | LI11 - SJ5 | 43 |
| 8 | Sanyinjiao(SP6) | 438 | LI15 - SJ5 | 314 | 8 | Shousanli(LI10) | 46 | LI4 - LR3 | 41 |
| 9 | Chize(LU5) | 321 | LI4 - GB34 | 313 | 9 | Yanglingquan(GB34) | 45 | LR3 - ST36 | 41 |
| 10 | Taichong(LR3) | 319 | LI4 - ST36 | 311 | 10 | Neiguan(PC6) | 37 | LI11 - LR3 | 40 |
| 11 | Neiguan(PC6) | 312 | LI11 - ST36 | 309 | 11 | Weizhong(BL40) | 34 | LI4 - LI10 | 39 |
| 12 | Xuehai(SP10) | 258 | LI10 - SJ5 | 303 | 12 | Chize(LU5) | 33 | LI15 - ST36 | 36 |
| 13 | Jiquan(HT1) | 225 | LI11 - GB34 | 287 | 13 | Jiquan(HT1) | 33 | LI4 - SP6 | 36 |
| 14 | Xuanzhong(GB39) | 220 | GB34 - ST36 | 281 | 14 | Baihui(DU20) | 26 | LI11 - SP6 | 36 |
| 15 | Jiexi(ST41) | 216 | LI15 - LI10 | 261 | 15 | Binao(LI14) | 24 | GB34 - ST36 | 36 |
| 16 | Yinlingquan(SP9) | 211 | LI4 - SP6 | 259 | 16 | Xuanzhong(GB39) | 23 | SJ5 - ST36 | 35 |
| 17 | Weizhong(BL40) | 204 | SJ5 - GB34 | 243 | 17 | Jiexi(ST41) | 22 | LI11 - LI10 | 35 |
| 18 | Huantiao(GB30) | 195 | SJ5 - ST36 | 241 | 18 | Anterior parietal temporal oblique line(MS6) | 19 | LI11 - GB34 | 35 |
| 19 | Baihui(DU20) | 173 | LI10 - GB34 | 240 | 19 | Fengshi(GB31) | 19 | SP6 - ST36 | 35 |
| 20 | Binao(LI14) | 145 | SP6 - ST36 | 233 | 20 | Huantiao(GB30) | 19 | LI15 - SJ5 | 34 |

| **Balance–gait disorder** | | | | |
| --- | --- | --- | --- | --- |
| **Sort** | **Acupoints** | **frequency** | **High-frequency Link** | **frequency** |
| 1 | Zusanli(ST36) | 80 | LI11 - ST36 | 50 |
| 2 | Quchi(LI11) | 61 | LI15 - ST36 | 49 |
| 3 | Yanglingquan(GB34) | 60 | GB34 - ST36 | 48 |
| 4 | Jianyu(LI15) | 58 | LI15 - GB34 | 47 |
| 5 | Sanyinjiao(SP6) | 53 | LI15 - LI11 | 47 |
| 6 | Hegu(LI4) | 52 | LI4 - LI15 | 45 |
| 7 | Shousanli(LI10) | 46 | LI4 - ST36 | 43 |
| 8 | Waiguan(SJ5) | 42 | LI4 - LI11 | 43 |
| 9 | Taichong(LR3) | 40 | LI11 - GB34 | 43 |
| 10 | Jiexi(ST41) | 38 | LI15 - LI10 | 40 |
| 11 | Anterior parietal temporal oblique line(MS6) | 37 | LI10 - ST36 | 40 |
| 12 | Baihui(DU20) | 35 | LI4 - GB34 | 39 |
| 13 | Biguan(ST31) | 31 | LI10 - GB34 | 38 |
| 14 | Liangqiu(ST34) | 31 | SP6 - ST36 | 37 |
| 15 | Motor area(Motor area) | 31 | LR3 - ST36 | 34 |
| 16 | Chize(LU5) | 29 | LI15 - SJ5 | 34 |
| 17 | Neiguan(PC6) | 27 | LI11 - LI10 | 33 |
| 18 | Shuigou(DU26) | 23 | LI11 - SJ5 | 33 |
| 19 | Binao(LI14) | 22 | SJ5 - GB34 | 33 |
| 20 | Weizhong(BL40) | 22 | ST41 - GB34 | 32 |
